# ADRB2 inhibition combined with antioxidant treatment alleviates lung fibrosis by attenuating TGFβ/SMAD signaling in lung fibroblasts

**DOI:** 10.1038/s41420-023-01702-9

**Published:** 2023-11-04

**Authors:** Ruyan Wan, Lan Wang, Yudi Duan, Miaomiao Zhu, Wenwen Li, Mengxia Zhao, Hongmei Yuan, Kai Xu, Zhongzheng Li, Xiao Zhang, Guoying Yu

**Affiliations:** 1 State Key Laboratory Cell Differentiation and Regulation, Henan International Joint Laboratory of Pulmonary Fibrosis, Henan center for outstanding overseas scientists of pulmonary fibrosis, College of Life Science, Institute of Biomedical Science, Henan Normal University, Xinxiang, Henan 453007 China; 2 Zhengzhou 101 Middle School, Zhengzhou, Henan 450000 China

**Keywords:** Molecular biology, Respiratory tract diseases

## Abstract

Idiopathic pulmonary fibrosis is a progressive and fatal interstitial lung disease with a poor prognosis and limited therapeutic options, which is characterized by aberrant myofibroblast activation and pathological remodeling of the extracellular matrix, while the mechanism remains elusive. In the present investigation, we observed a reduction in ADRB2 expression within both IPF and bleomycin-induced fibrotic lung samples, as well as in fibroblasts treated with TGF-β1. ADRB2 inhibition blunted bleomycin-induced lung fibrosis. Blockage of the ADRB2 suppressed proliferation, migration, and invasion and attenuated TGF-β1-induced fibroblast activation. Conversely, the enhancement of ADRB2 expression or functionality proved capable of inducing fibroblast-to-myofibroblast differentiation. Subsequent mechanistic investigation revealed that inhibition of ADRB2 suppressed the activation of SMAD2/3 in lung fibroblasts and increased phos-SMAD2/3 proteasome degradation, and vice versa. Finally, ADRB2 inhibition combined with antioxidants showed increased efficacy in the therapy of bleomycin-induced lung fibrosis. In short, these data indicate that ADRB2 is involved in lung fibroblast differentiation, and targeting ADRB2 could emerge as a promising and innovative therapeutic approach for pulmonary fibrosis.

## Introduction

Idiopathic pulmonary fibrosis (IPF) is a chronic and progressive interstitial lung disease that is characterized by excessive fibroblast activation and extensive accumulation of extracellular matrix, ultimately leading to the deterioration of lung function [1–4]. The median survival is estimated to be 2–4 years, and the incidence of IPF increases with age [5]. Epidemiological studies have suggested that multiple risk factors, including environmental risk factors [6–8], genetic susceptibility [9–11], and virus infections [12, 13], enhance susceptibility to the disease [14]. To date, the U.S. FDA has granted approval for only two medicines, namely pirfenidone and nintedanib, in the therapeutic management of IPF [15]. While these medications have demonstrated a certain degree of efficacy in retarding the progression of the disease, their comprehensive mechanisms remain inadequately comprehended, and they are associated with adverse effects. Therefore, studying the process and regulation of pulmonary fibrosis is crucial for the diagnosis and therapy of IPF.

Although the causes of pulmonary fibrosis are multifarious, research has shown that fibroblasts differentiate into myofibroblasts and subsequently produce excess extracellular matrix proteins with transforming growth factor β1 (TGF-β1) as one of the key mediators [16]. Thus, the identification of additional novel mediators that inhibit the TGF-β signaling pathway is urgently needed to alleviate lung fibrosis.

Emerging studies have shown that increased oxidative stress appears to drive the progression of IPF by various mechanisms [17–19]. Indeed, an imbalance between endogenous antioxidants and oxidants is considered a typical feature of IPF [20]. For example, reactive oxygen species (ROS) activate the TGF-β/SMAD signaling pathway and mediate many profibrogenic effects [21]. Nonetheless, the potential for antioxidant monotherapy to effectively reverse the progression of IPF is unlikely due to the restricted effectiveness exhibited by antioxidants [22]. This suggests that the combination of antioxidant agents and other antifibrotic agents may be a useful therapeutic approach.

G protein-coupled receptors (GPCRs) comprise an expansive and different family of proteins that primarily serve to convert extracellular stimuli into intracellular signals [23]. It has been estimated that GPCRs represent ~34% of the molecular targets for U.S. FDA-approved drugs [24]. Lately, numerous pairs of GPCR ligands and receptors have been identified as potential drivers for aberrant fibroblast activation in vitro and contributors to the development of fibrosis in vivo. [23]. For example, in a bleomycin-induced lung fibrosis mouse model, lysophosphatidic acid receptor 1 (LPA1) deficiency resulted in a reduced level of fibroblast recruitment [25]. The administration of LPA1 inhibitor BMS-986020 in patients with IPF can effectively slow the decline in force vital capacity [26].

Adrenoceptor beta 2 (ADRB2), a member of the GPCRs, has been implicated in the underlying mechanisms of organ fibrosis. In the heart, ADRB2 activation prevents cardiac fibrosis by reducing collagen production and increasing collagen degradation of cardiac fibroblasts [27, 28]. Moreover, ADRB2 agonizts can significantly reduce human dermal fibroblast differentiation and contractile function by multiple mechanisms and inhibit the expression of profibrotic markers [29]. Recent literature has revealed that ADRB2 is widely distributed in the respiratory system [30–32]. However, the precise role of ADRB2 in the activation of resident fibroblasts and the development of pulmonary fibrosis remains understudied. Moreover, the abnormal expression of ADRB2 within our comprehensive dataset of fibrotic lungs prompted us to investigate its potential involvement in fibrosis progression. Our research aimed to elucidate the role of ADRB2 in fibroblast activation and matrix production and the feasibility of ADRB2 as an alternative therapeutic target in IPF.

## Results

### ADRB2 was decreased in fibrotic lungs and TGF-β1-stimulated fibroblasts

**Fig. 1:**
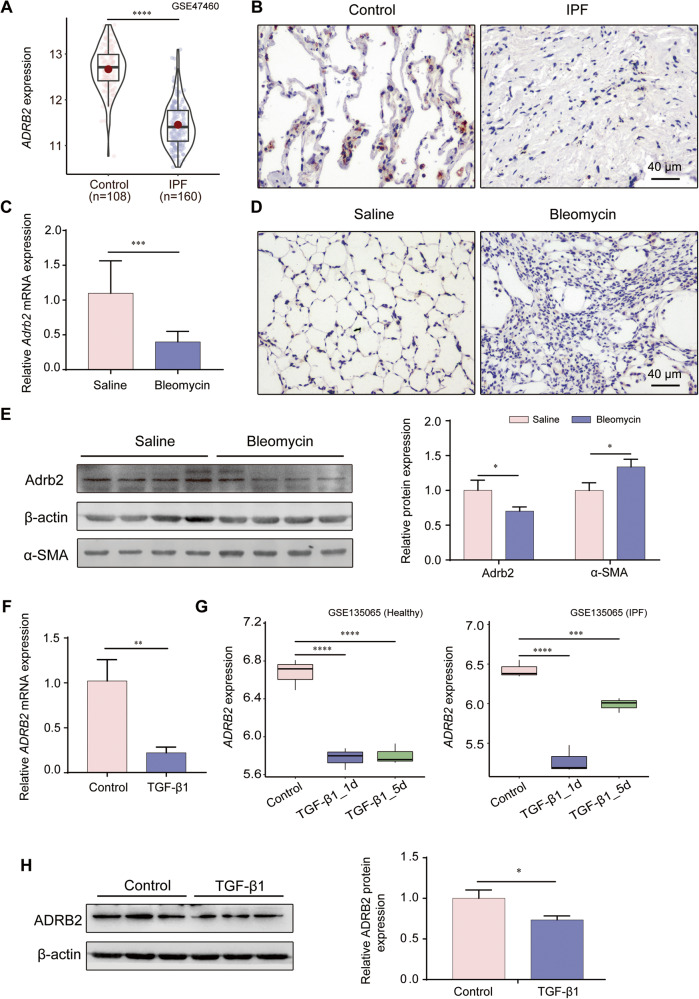
Decreased ADRB2 expression in fibrotic lungs and TGF-β1 stimulated lung fibroblasts. **A** Transcriptional change of the *ADRB2* gene was identified in independent RNAseq (GSE47460). **B** Representative images of ADRB2 IHC staining on lung sections from IPF patients and control subjects (*n* = 3). **C** The transcriptional change of *Adrb2* of saline- and bleomycin-induced mice by qRT-PCR analysis (*n* = 3). **D** Representative images of Adrb2 IHC staining on lung sections from bleomycin-induced mice and saline control (*n* = 3). **E** The protein expression of Adrb2 and α-SMA of saline- and bleomycin-induced mice by WB analysis (*n* = 4). **F** qRT-PCR analysis of TGF-β1-induced ADRB2 expression in MRC-5 cells (*n* = 3). **G** *ADRB2* mRNA expression was downregulated in TGF-β1-stimulated primary human lung fibroblasts from control subjects or IPF patients. Reanalyzes of publicly available microarray data (GSE135065) in TGF-β1-stimulated primary human lung fibroblasts from control subjects and IPF patients. Data are presented as Box plots. Unpaired *t*-test and variance analysis with Holm’s adjustment for independent samples. *** adjusted-*P*value < 0.001; **** adjusted-*P*value < 0.0001. **H** WB analysis of ADRB2 expression in MRC-5 cells treated for 48 h with and without TGF-β1 (10 ng/mL) (*n* = 3). Data are shown as the mean ± SD. *P < 0.05; ** <0.01; ***P < 0.001; ****P < 0.0001.

In order to investigate the plausible role of ADRB2 in pulmonary fibrosis, we conducted an examination of ADRB2 expression in fibrotic lungs. Analysis of the Gene Expression Omnibus (GEO) database (GSE47460) revealed that *ADRB2* mRNA levels were significantly decreased in IPF lungs compared with control (Fig. 1A). Histological examination of IPF lungs showed decreased ADRB2 protein expression compared with that of the control (Fig. 1B). Consistent with these research results, reanalyzes of publicly available microarray data also demonstrated downregulation of *ADRB2* mRNA expression in the IPF lung samples (GSE32537, GSE24206, GSE110147, GSE124685, Supplementary Fig. 1A). In parallel, the lung tissues of mice treated with bleomycin exhibited notably reduced expression of Adrb2 in comparison to control mice. This was accompanied by a pronounced elevation in the level of the fibrotic marker α-SMA, specifically observed 21 days after bleomycin treatment. Furthermore, immunohistochemical staining highlighted a significant decrease in the expression of Adrb2 within fibrotic regions of bleomycin-exposed mice when contrasted with control counterparts (Fig. 1C–E). TGF-β1 downregulated ADRB2 expression in MRC-5 and IMR-90 cells at both the transcriptional and protein levels, as shown in Fig. 1F, H and Supplementary Fig. 2A, B. This was reinforced by reanalyzes of microarray data on primary lung fibroblasts from control or IPF showed that *ADRB2* mRNA level was significantly downregulated at day 1 and day 5 post TGF-β1 stimulation (GSE 135065, Fig. 1G). Meanwhile, we found that phos-SMAD2/3 expression was significantly increased in bleomycin-treated fibrotic mouse lung tissues and TGF-β1-stimulated lung fibroblasts compared with controls (Supplementary Fig. 2C, D).

### ADRB2 inhibition ameliorated bleomycin-induced pulmonary fibrosis in mice

**Fig. 2:**
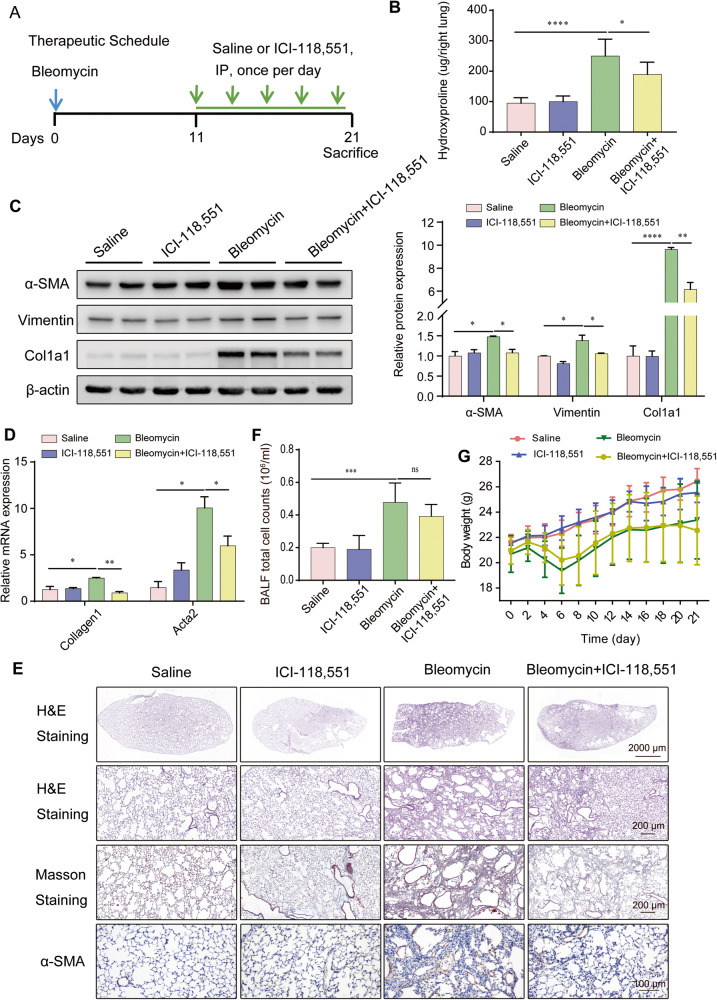
Pharmacologic inhibition of ADRB2 ameliorated bleomycin-induced pulmonary fibrosis in mice. **A** A diagram depicting the experimental procedure for the in vivo administration of bleomycin and ICI-118,551. **B**–**D** Effect of saline and ICI-118,551 treatment in mice challenged with bleomycin or not, as reflected by values measured at day 21 for the right lung hydroxyproline content (**B**, Saline *n* = 7, ICI-118,551 *n* = 7, Bleomycin *n* = 7, Bleomycin+ICI-118,551 *n* = 7), protein level of the fibrotic markers α-SMA, Vimentin and Col1a1 (**C**, *n* = 3), and mRNA expression of the myofibroblast markers *Acta2* and *Col1a1* (**D**, *n* = 3). **E** Digital photomicrographs of H&E, Masson’s trichrome, and IHC staining analysis of α-SMA staining from each group of treated mice at day 21 (*n* = 3). **F** Quantification of the total number of inflammatory cells in BALF from each group of the experimental animals (Saline *n* = 5, ICI-118,551 *n* = 5, Bleomycin *n* = 5, Bleomycin+ICI-118,551 *n* = 5). **G** Body weight was monitored throughout the experiment (Saline *n* = 10, ICI-118,551 *n* = 10, Bleomycin *n* = 10, Bleomycin+ICI-118,551 *n* = 10). Data are shown as the mean ± SD. *P < 0.05; ** <0.01; ***P < 0.001.

To examine the function of Adrb2 on the process of fibrogenesis in vivo, a mouse model of pulmonary fibrosis was established using intratracheal administration of bleomycin. Subsequent to a solitary administration of bleomycin (1.5 U/kg) on Day 0, mice were subjected to daily intraperitoneal injections of the ADRB2 selective inhibitor ICI-118,551 (2 mg/kg) or saline for a duration of 10 days. On Day 21, mouse lungs were collected (Fig. 2A). Treatment with ICI-118,551 significantly blunted the fibrosis by decreasing the hydroxyproline content in mice (Fig. 2B). Bleomycin treatment significantly increased the protein level of Col1a1, α-SMA, and Vimentin. The Adrb2 inhibitor ICI-118,551 significantly attenuated bleomycin-induced mouse lung fibrosis (Fig. 2C). At the RNA level, *Col1a1* and *Acta2* expression was significantly suppressed by ICI-118,551 (Fig. 2D). Furthermore, administration of ICI-118,551 dramatically reduced bleomycin-induced lung fibrosis as demonstrated by H&E, Masson’s staining and immunohistochemical analysis of α-SMA (Fig. 2E). There was no notable difference in inflammatory cell counts in the bronchoalveolar lavage (BAL) fluid or body weight between the saline and ICI-118,551 treatment groups after bleomycin challenge (Fig. 2F, G). Together, these results demonstrate that inhibition of Adrb2 with ICI-118,551 substantially attenuates bleomycin-induced pulmonary fibrosis.

### ADRB2 was critical for fibroblast proliferation, migration, and activation

**Fig. 3:**
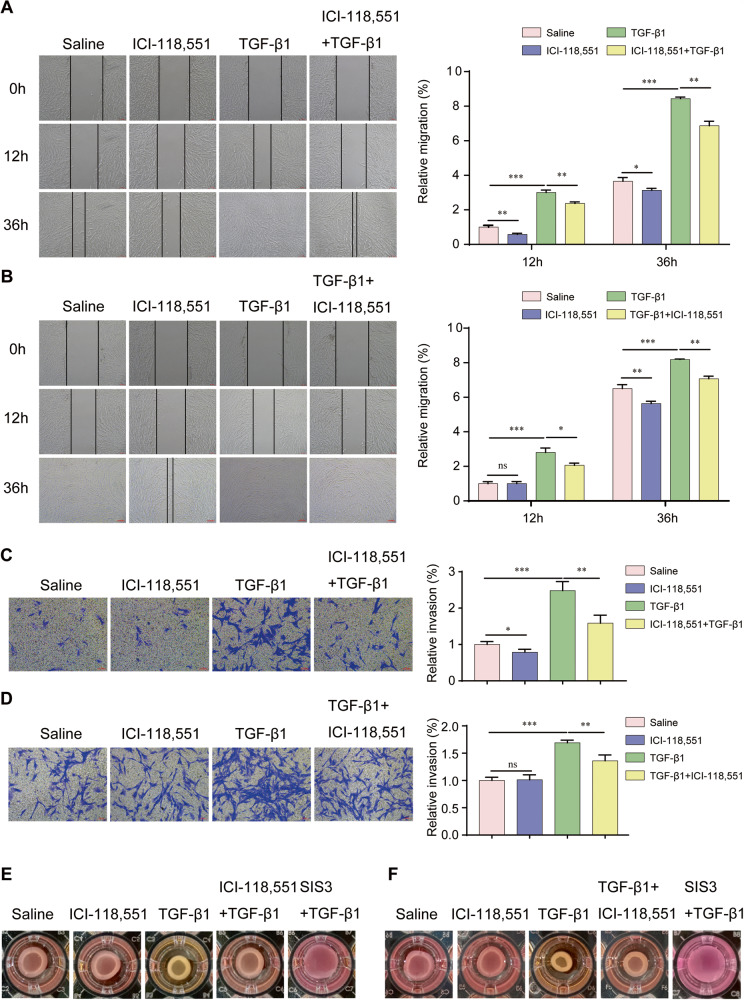
ADRB2 inhibition suppressed the migration and activation of fibroblasts. **A**, **B** The migration of MRC-5 cells in different treatment groups was tested by the scratch wound assay. Cells were treated with 20 μM ICI-118,551 for 24 h, then stimulated with TGF-β1 (10 ng/mL) for 24 h (**A**). Cells were treated with 10 ng/mL TGF-β1 for 24 h, then stimulated with 20 μM ICI-118,551 for 24 h (**B**) (*n* = 3). **C**, **D** Transwell assay was used to detect the invasion ability of MRC-5 cells. Cells were treated with 20 μM ICI-118,551 for 24 h prior to challenge with 10 ng/mL TGF-β1 (C, *n* = 3). Cells were treated with 10 ng/mL TGF-β1 for 24 h, then stimulated with 20 μM ICI-118,551 for 24 h (**D**, *n* = 3). **E**, **F** Collagen contraction assay was used to evaluate the myofibroblast activation in different treatment groups. MRC-5 cells were treated with 20 μM ICI-118,551 for 24 h, then stimulated with TGF-β1 (10 ng/mL) for 24 h (**E**, *n* = 3). MRC-5 cells were treated with 10 ng/mL TGF-β1 for 24 h, then stimulated with 20 μM ICI-118,551 for 24 h (**F**, *n* = 3). Data are shown as the mean ± SD. *P < 0.05; ** <0.01; ***P < 0.001.

To gain deeper insights into the functional significance of ADRB2, we performed in vitro assays using lung fibroblasts. The administration of the ADRB2 inhibitor ICI-118,551 significantly reduced the cell viability, proliferative activity, and protein expression of TGF-β1 in MRC-5 cells, while the activation of ADRB2, cell viability, proliferative activity, and protein expression of TGF-β1 in MRC-5 cells was significantly enhanced (Supplementary Fig. 3A–E). To investigate whether ADRB2 affects lung fibroblast behaviors, we measured the migration and invasion of MRC-5 cells after ICI-118,551 treatment and found that the administration of TGF-β1 led to an augmentation in the migration and invasion capabilities of MRC-5 cells, and this enhancement was partially suppressed or reversed by ADRB2 inhibition (Fig. 3A–D). In contrast, activation or overexpression of ADRB2 increased the migration and invasion of MRC-5 cells, both in the presence and absence of TGF-β1 (Supplementary Fig. 4A–D and Supplementary Fig. 5A). In summary, these findings demonstrate that ADRB2 is vital for promoting the proliferation, migration, and invasion of lung fibroblasts.

To determine whether the inhibition of ADRB2 alters collagen contraction, we performed collagen gel contraction assays and showed that TGF-β1-induced collagen gel contraction was inhibited by ICI-118,551 at 18% in MRC-5 cells (Fig. 3E, Supplementary Fig. 3F). After preincubation of MRC-5 cells with TGF-β1, followed by treatment with ICI-118,551, the size of the collagen gel lattices increased compared to those treated solely with TGF-β1 (Fig. 3F, Supplementary Fig. 3F). These results indicated that inhibition of ADRB2 blunted TGF-β1-stimulated contraction of collagen gel in MRC-5 cells, regardless of whether they were first treated with TGF-β1 and ICI-118,551. Conversely, activation of ADRB2 further augmented TGF-β1-stimulated contraction in MRC-5 cells (Supplementary Fig. 3F and Supplementary Fig. 4E, F).

### ADRB2 inhibition prevented TGF-β1-induced myofibroblast differentiation

**Fig. 4:**
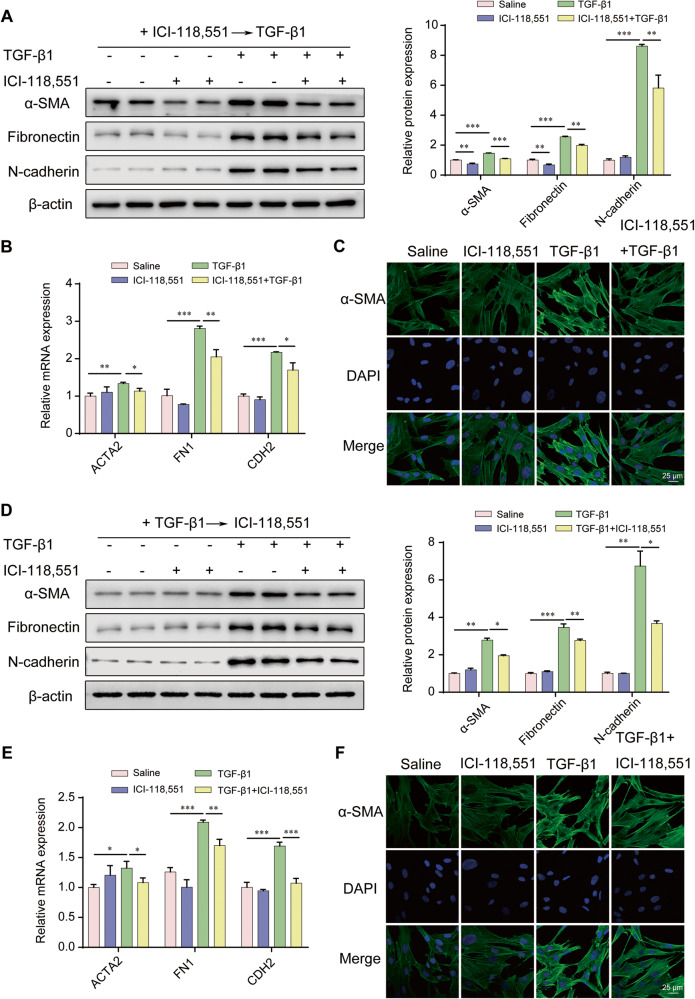
Pharmacologic inhibition of ADRB2 dampened TGF-β1 induction of fibroblast to myofibroblast differentiation. **A**, **B** Effects of 24 h pretreatment of MRC-5 cells with 20 μM ICI-118,551 on TGF-β1–induced expression of α-SMA, Fibronectin and N-cadherin protein (**A**) and mRNA (**B**) (prevention protocol) (*n* = 3). **C** Representative ICC staining of α-SMA expression in MRC-5 cells was treated with 20 μM ICI-118,551 for 24 h, prior to challenge with TGF-β1 (10 ng/mL) (*n* = 3). **D** WB and **E** qRT-PCR analysis of α-SMA, Fibronectin, and N-cadherin expression in TGF-β1-generated myofibroblasts treated with 20 μM ICI-118,551 for 24 h (reversal protocol) (*n* = 3). **F** ICC analysis of α-SMA expression in TGF-β1-induced myofibroblasts treated with 20 μM ICI-118,551 for 24 h. Data are shown as the mean ± SD. *P < 0.05; ** <0.01; ***P < 0.001.

We next assessed the role of ADRB2 inhibition on fibroblast differentiation by treating MRC-5 cells with ICI-118,551 combined with TGF-β1 stimulation. Pretreatment of MRC-5 cells with ICI-118,551 (20 μM) markedly prevented TGF-β1-induced myofibroblast differentiation (Fig. 4A–C). Notably, inhibition of ADRB2 blunted α-SMA, Collagen 1 and N-cadherin protein expression in the IMR-90 cells, irrespective of the presence or absence of TGF-β1 stimulation (Supplementary Fig. 6A). The same results regarding this reaction were also observed under TGF-β1-induced primary mouse lung fibroblasts (Supplementary Fig. 6B). In addition, ICI-118,551 treatment significantly down-regulated the level of α-SMA, Fibronectin, and N-cadherin in myofibroblasts, which had undergone differentiation from fibroblasts following prior exposure to TGF-β1 (Fig. 4D–F, Supplementary Fig. 6C, D). Together, these data indicate that inhibition of ADRB2 attenuated TGF-β1-induced myofibroblast differentiation.

### Activation of ADRB2 was sufficient to trigger fibroblast activation and ECM production

Next, we investigated the effect of increased ADRB2 expression or activity on ECM production in fibroblasts. Overexpression of ADRB2 in MRC-5 cells led to remarkable upregulation of TGF-β1-induced profibrotic markers, including α-SMA, Fibronectin and N-cadherin, at both the protein and transcriptional levels compared to mock transfection in MRC-5 cells (Supplementary Fig. 6B, C). Similar results were observed in the presence of 20 μM epinephrine, an ADRB2 agonist (Supplementary Fig. 7A–C). Consistent with the findings in MRC-5 cells, epinephrine also increased TGF-β1-induced IMR-90 cells and primary mouse lung fibroblast cell activation (Supplementary Fig. 8A, B). Because epinephrine is not specific to ADRB2, we further demonstrated that epinephrine treatment promotes fibroblast-to-myofibroblast differentiation in this study by activating ADRB2 rather than other adrenergic receptors. As shown in Supplementary Fig. 8C, stimulation of MRC-5 cells with epinephrine enhanced TGF-β1-induced profibrotic protein expression, including that of α-SMA and N-cadherin. This augmentation was inhibited through prior treatment with the ADRB2 highly selective inhibitor ICI-118,551. These results demonstrate that epinephrine treatment promotes fibroblast-to-myofibroblast differentiation come from the activation of ADRB2. Furthermore, epinephrine treatment up-regulated the expression of α-SMA, Fibronectin and N-cadherin in myofibroblasts that pretreat with TGF-β1 first (Supplementary Fig. 7D-F). In summary, these data revealed that the gain of ADRB2 expression or activity in both IMR-90 and MRC-5 cells was sufficient to promote fibroblast-to-myofibroblast differentiation and ECM production.

### ADRB2-induced profibrotic responses were mediated through TGFβ/SMAD signaling

**Fig. 5:**
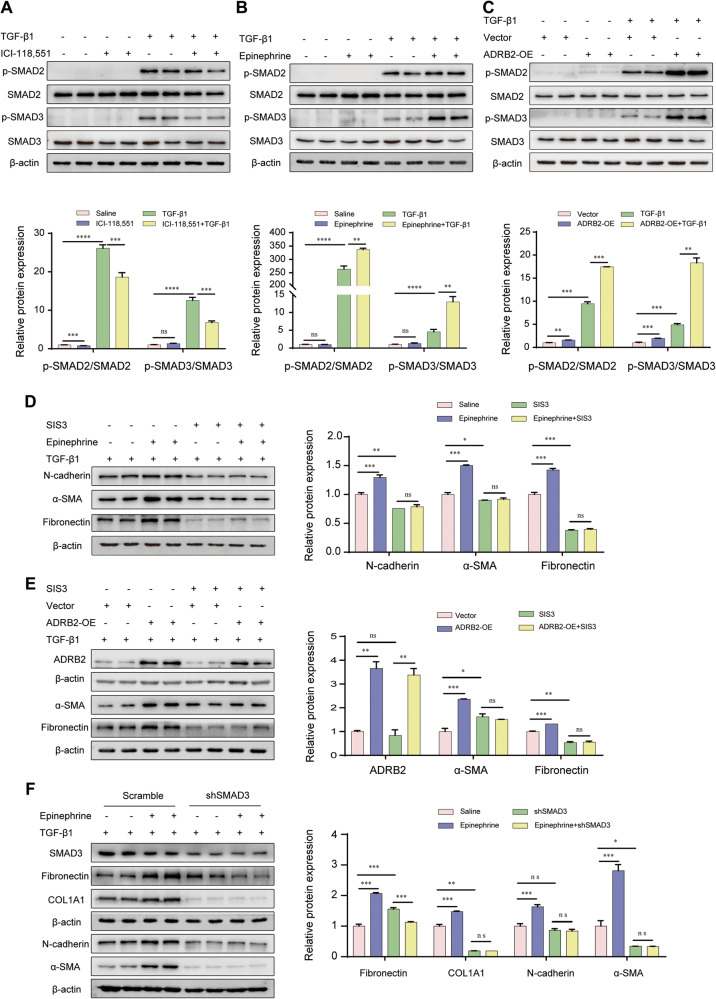
ADRB2-induced profibrotic responses are mediated through TGF-β/SMADs signaling. **A**, **B** Representative WB analysis of SMAD2, SMAD3 expression and their phosphorylated forms in MRC-5 cells treated with or without 20 μM ICI-118,551 (**A**) or 20 μM epinephrine (**B**) for 24 h, prior to challenge with TGF-β1 (10 ng/mL) (*n* = 3). **C** Protein expression levels of SMAD2, SMAD3, and their phosphorylated forms in MRC-5 cells transfected with ADRB2 plasmid or empty control for 24 h, prior to challenge with TGF-β1 (10 ng/mL) (*n* = 3). **D**, **E** Cells were treated with 20 μM epinephrine or overexpression of ADRB2 for 24 h in the presence or absence of SMAD3 inhibitor ((E)-SIS3) for 2 h, then cells were treated with TGF-β1 (10 ng/mL) for 24 h. The expression of α-SMA, Fibronectin, N-cadherin, and ADRB2 was determined by WB analysis (*n* = 3). **F** WB analysis of the effect of shSMAD3 or mock on TGF-β1-induced α-SMA, COL1A1, Fibronectin, and N-cadherin in MRC-5 cells that were treated with 20 μM epinephrine or saline for 48 h (*n* = 3). Data are shown as the mean ± SD. *P < 0.05; ** <0.01; ***P < 0.001.

Because ADRB2 was essential for TGF-β1-induced lung fibroblast activation and ECM production, we tested the ADRB2 regulation of TGF-β1 signaling. ICI-118,551 treatment partially inhibited the phosphorylation of SMAD2/3 triggered by TGF-β1 stimulation in MRC-5 cells and primary mouse lung fibroblast cells (Fig. 5A, Supplementary Fig. 9A) and promoted its nuclear export (Supplementary Fig. 9B, C). In contrast, activation or overexpression of ADRB2 further increased the expression of TGF-β1-induced phos-SMAD2/3 in MRC-5 cells (Fig. 5B, C). These findings were confirmed by immunofluorescence analysis (Supplementary Fig. 9D, E). Notably, immunoblotting and IHC also showed markedly attenuated upregulation of phos-Smad2/3 expression in the ICI-118,5551-treated mice compared with the control mice upon challenge with bleomycin (Supplementary Fig. 10A, B). These results indicate that ADRB2 potentially modulates the activation of myofibroblasts by exerting regulatory control over the TGFβ/SMAD signaling pathway.

To further determine whether ADRB2 regulates myofibroblast activation by regulating SMAD signaling, we inhibited SMAD3 in MRC-5 cells with (E)-SIS3, followed by TGF-β1 stimulation. Stimulation of MRC-5 cells with epinephrine or overexpression of ADRB2 enhanced TGF-β1-induced profibrotic protein expression, including that of α-SMA, Fibronectin, and N-cadherin. This augmentation was impeded through prior treatment with the SMAD3 inhibitor (E)-SIS3 (Fig. 5D, E). Similar results were also observed after knocking down SMAD3 expression with shRNA in MRC-5 cells (Fig. 5F).

**Fig. 6:**
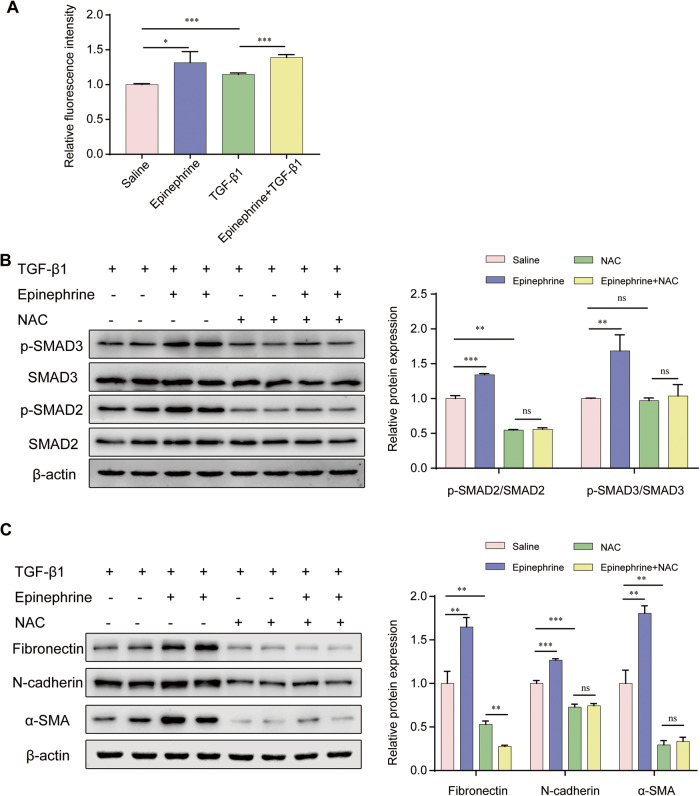
ADRB2 modulated TGF-β/SMADs signaling via ROS. **A** MRC-5 cells were pretreated with pharmacologic agonist epinephrine (20 μM) and then stimulated with TGF-β1 (10 ng/ml) for 24 h. The intracellular ROS level was assessed by the dihydroethidium assay (*n* = 3). **B** Levels of SMAD2, SMAD3, and their phosphorylated forms in MRC-5 cells treated with or without 20 μM epinephrine for 24 h in the presence or absence of ROS scavenger NAC (5 mM), prior to challenge with TGF-β1 (10 ng/mL) were determined by WB analysis (*n* = 3). **C** MRC-5 cells were treated with 20 μM epinephrine for 24 h in the presence or absence of NAC (5 mM), then cells were treated with TGF-β1 (10 ng/mL) for 24 h. The protein expression of α-SMA, Fibronectin, and N-cadherin was determined by WB analysis (*n* = 3). Data are shown as the mean ± SD. *P < 0.05; **P < 0.01; ***P < 0.001.

With respect to the altered SMAD2/3 signaling pathway, we further hypothesized that ADRB2 may modulate TGF-β1 signaling *via* a secondary messenger. As predicted, the level of ROS was significantly elevated after epinephrine stimulation in MRC-5 cells regardless of TGF-β1 induction (Fig. 6A). To determine whether ADRB2 regulates the SMAD2/3 signaling pathway by altering ROS levels, we evaluated the phosphorylation of SMAD2/3 after ROS elimination. As shown in Fig. 6B, stimulation of MRC-5 cells with epinephrine enhanced TGF-β1-induced phosphorylation of SMAD2/3. This increase was blocked by pretreatment with the ROS scavenger N-acetyl cysteine (NAC). In addition, we found that the TGF-β1- stimulated increases in α-SMA, fibronectin, and N-cadherin protein expression in MRC-5 cells were also suppressed by NAC (Fig. 6C). These results indicate that ADRB2-mediated ROS production has a signaling role that contributes to the activation of SMAD2/3.

### ADRB2 increased phos-SMAD2/3 expression by enhancing phos-SMAD2/3 protein stability

**Fig. 7:**
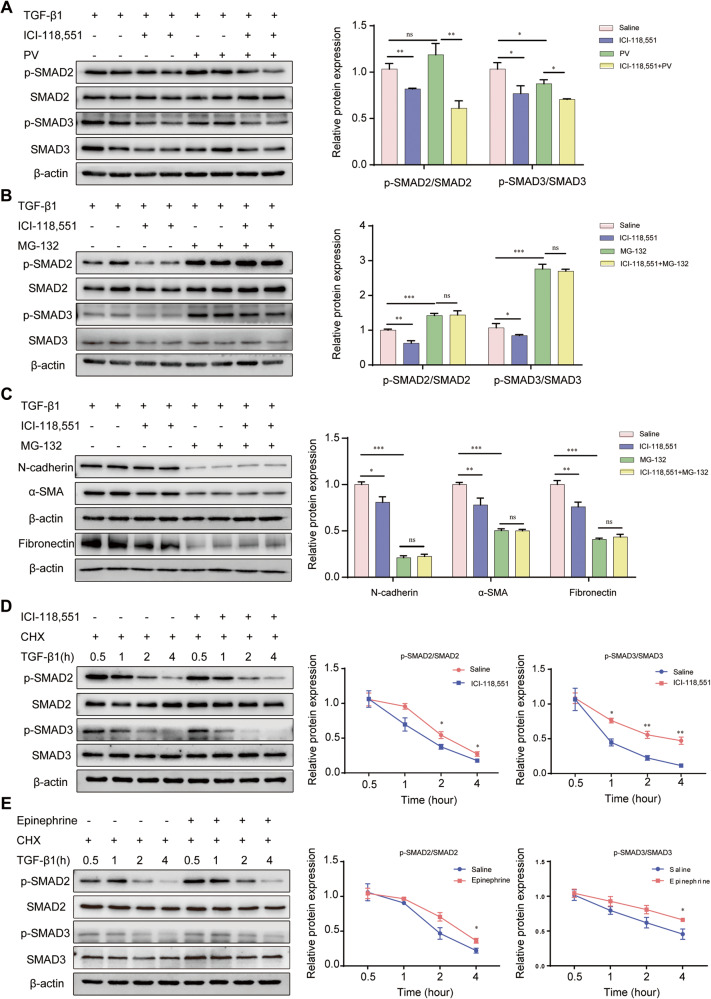
ADRB2 enhanced the protein stability of SMAD2/3 expression in lung fibroblasts. **A**, **B** WB analysis of TGF-β1-induced SMAD2/3 phosphorylation in MRC-5 cells that were pre-treated with 20 μM ICI-118,551 for 24 h, followed with a pre-treatment for 2 h with 100 μg/ml pervanadate (**A**) or 20 μM MG-132 (**B**) before stimulation with 10 ng/mL TGF-β1 (*n* = 3). **C** The protein expression of α-SMA, Fibronectin, and N-cadherin in MRC-5 cells that were pre-treated with 20 μM ICI-118,551 for 24 h, followed with a pre-treatment for 2 h with 20 μM MG-132 before stimulation with 10 ng/mL TGF-β1 (*n* = 3). **D**, **E** Western blotting was performed to measure SMAD2/3 and phos-SMAD2/3 protein degradation after treatment with 100 μg/ml CHX for different lengths of time in MRC-5 cells that pre-treatment with 20 μM ICI-118,551 (**D**) or 20 μM epinephrine (**E**) for 24 h (*n* = 3). Data are shown as the mean ± SD. *P < 0.05; **P < 0.01; ***P < 0.001.

To determine how treatment with the ADRB2 inhibitor ICI-118,551 might affect the steady-state level of phos-SMAD2/3, we pretreated MRC-5 cells with the wide-spectrum phosphatase inhibitor pervanadate or the proteasome inhibitor MG-132 for 2 h before stimulation with TGF-β1. The addition of pervanadate did not affect the loss of TGF-β1-induced SMAD2/3 phosphorylation (Fig. 7A). In contrast, the use of MG-132 markedly abrogated the TGF-β1-dependent decrease in phos-SMAD2/3 expression in the ICI-118,551-treated MRC-5 cells (Fig. 7B). ADRB2 inhibition-induced TGF-β1-dependent α-SMA, Fibronectin and N-cadherin downregulation in MRC-5 cells was rescued with MG-132 treatment (Fig. 7C). These data suggest that ADRB2 enhanced phos-SMAD2/3 expression by, at least partially, stabilizing the phos-SMAD2/3 protein.

Furthermore, to determine how ADRB2 regulates phos-SMAD2/3 protein expression in lung fibroblasts, we performed a cycloheximide (CHX) protein chase assay to evaluate the protein stability of phos-SMAD2/3 in the MRC-5 cells treated with an ADRB2 inhibitor or agonist. As shown in Fig. 7D, with the protein synthesis inhibitor CHX, inhibition of ADRB2 led to accelerated degradation of phos-SMAD2/3 in MRC-5 cells. However, activation of ADRB2 increased phos-SMAD2/3 protein stability (Fig. 7E). Inhibition of ADRB2 enhanced the ubiquitination of phos-SMAD3 and vice versa (Supplementary Fig. 11A, B). In vitro ubiquitination assays with ubiquitin mutants (K48O and K63O) indicated that ADRB2 mediated K48-linked ubiquitination of phos-SMAD3 (Supplementary Fig. 11C, D). Putting together, we conclude that ADRB2 maintains the stability of the phos-SMAD2/3 protein in lung fibroblasts and that a lack of ADRB2 promotes the degradation of phos-SMAD2/3 by the proteasome.

### ADRB2 inhibition combined with NAC increased efficacy in the therapy of lung fibrosis

**Fig. 8:**
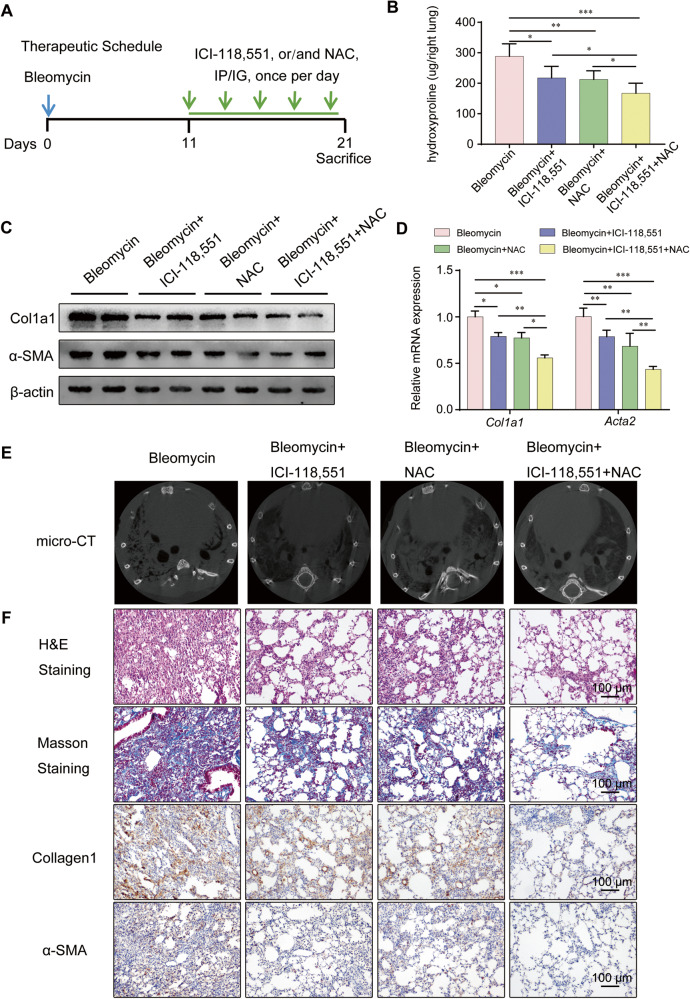
Pharmacologic inhibition of ADRB2 combined with NAC increased efficacy in the treatment of bleomycin-induced lung fibrosis. **A** Schematic illustrating the experimental protocol for bleomycin-induced pulmonary fibrosis in mice treated with saline, ICI-118,551 (2 mg/kg) only, NAC (500 mg/kg) only, or ICI-118,551 plus NAC. **B** Collagen deposition evaluated by hydroxyproline content per right lung in bleomycin-exposed mice after treatment with saline, ICI-118,551 only, NAC only, and ICI-118,551 combined with NAC (Saline *n* = 6, ICI-118,551 *n* = 6, Bleomycin *n* = 6, Bleomycin+ICI-118,551 *n* = 6). **C** Protein expression levels of α-SMA and Col1a1 were analyzed by WB in lungs from bleomycin-challenged mice after various treatments (*n* = 3). **D** qRT-PCR analysis of relative change in *Acta2* and *Col1a1* mRNA levels in bleomycin-exposed mice after treatment with saline, ICI-118,551 only, NAC only, and ICI-118,551 combined with NAC (*n* = 3). **E** Representative micro-CT images of the lungs. Axial images of bleomycin-challenged mice were acquired at 21 days after saline, ICI-118,551 only, NAC only, or ICI-118,551 plus NAC treatments. **F** Representative photomicrographs of H&E-stained, Masson’s trichrome-stained, and α-SMA and Collagen1 IHC staining on lung sections from mice given ICI-118,551 and/or NAC. Data are shown as the mean ± SD. *P < 0.05; ** <0.01, ***P < 0.001.

Combination therapy of ICI-118,551 (2 mg/kg, i.p.) with NAC (500 mg/kg, i.g.) was performed as Fig. 8A, and hydroxyproline in the whole right lung was marked decreased as shown in Fig. 8B in comparison to saline or monotherapy only. The protein and RNA expressions of Col1a1 and α-SMA in the lungs were significantly lower (Fig. 8C, D). Micro-CT images taken 21 days after bleomycin exposure mice revealed a marked alteration in the lung density, indicated by heightened parenchymal opacity. The mice treated with ICI-118,551 or NAC had improved lung architecture compared with the mice exposed to bleomycin alone, while combination therapy exhibited the best therapeutic effect significantly (Fig. 8E). These differences in the destruction of lung architecture and collagen accumulation were further confirmed by H&E staining, Masson’s trichrome staining and immunohistochemical analysis of α-SMA and Collagen 1 of the lung sections, which demonstrated marked decreased collagen deposition in the ICI-118,551 combined with NAC therapy group compared with the monotherapy groups (Fig. 8F). These data suggested that Adrb2 inhibition with ICI-118,551 can be a therapy for pulmonary fibrosis, while combination with NAC increased the efficacy of pulmonary fibrosis.

## Discussion

IPF is a chronic lung disease of unknown etiology marked by substantial accumulation of ECM and diminished pulmonary capacity [33–35]. Despite the demonstrated ability of pirfenidone and nintedanib to decelerate the advancement of the disease, additional research is necessary to enhance the comprehension of prognosis and therapeutic approaches for individuals afflicted with IPF [36]. Identification of new targets for the regulation of fibroblast activation and ECM production is urgently needed. The study reported here helps elucidate a new role of ADRB2 and ADRB2 regulation of TGF-β/SMAD signaling, which is vital in facilitating fibrosis. We demonstrated that ADRB2 is downregulated in both IPF patients and the bleomycin model. ADRB2 inhibition in vitro and in vivo attenuated fibroblast proliferation, migration, activation, and ECM production under pro-fibrotic conditions (e.g., TGF-β1 or bleomycin exposure), indicating a destructive function of ADRB2 in lung fibrosis. This phenomenon is similar to the protective upregulation of proteins in pathological states. For example, TRIM33 is upregulated in IPF patients and bleomycin-challenged mice. However, downregulation of TRIM33 promoted the TGF-β1 signaling pathway and aggravated lung fibrosis [37]. In addition, increased lysocardiolipin acyltransferase (LYCAT) was observed in fibrotic lungs. LYCAT overexpression reduced bleomycin- and radiation-induced pulmonary fibrosis [38]. Hence, under pro-fibrotic conditions, the downregulation of ADRB2 may be a protective mechanism to prevent pulmonary fibrosis. Further investigation revealed that inhibition of ADRB2 impedes lung fibrosis by reducing TGF-β/SMAD signaling activity through the acceleration of phos-SMAD2/3 proteasome degradation. The present study shows ADRB2 downregulation has a protective effect against lung fibrosis through ADRB2 regulation of TGF-β signaling. We provide a proof of concept for the utilization of ICI-118,551 combined with NAC, which showed enhanced effectiveness in the treatment of bleomycin-induced lung fibrosis, leading to mitigated fibrotic progression.

Myofibroblasts are key effectors during wound healing and tissue repair due to their high contractility. Recent investigations employing lineage tracing to delve into the source of myofibroblasts in lung fibrosis have revealed that there is a notable absence or minimal occurrence of epithelial to mesenchymal transition [39] and that collagen1-expressing resident lung fibroblasts are a main source of myofibroblasts [40, 41]. However, continuous activation of myofibroblasts can lead to tissue dysfunction and disease [42, 43]. TGF-β1 is a prime cytokine involved in myofibroblast differentiation [44]. Activation of ADRB2 stimulated proliferation, promoted migration, and increased the contractility of MRC-5 cells under both control and TGF-β1 stimulation in vitro, all consistent with the induction of activated lung fibrosis [45, 46]. In contrast, inhibition of ADRB2 by ICI-118,551 inhibited and reversed TGF-β1 induction of myofibroblast differentiation. Overall, the observed ADRB2-mediated fibroblast responses paved the way for a better understanding of how ADRB2 facilitates lung fibrosis.

ECM remodeling, the result of fibrosis and a driving factor of fibrosis, is a highly dynamic process used to fine-tune physiological functions, including tissue homeostasis and damage repair [47, 48]. A large number of ECM components (such as collagens and fibronectin) are produced by myofibroblasts [49]. Both activation and overexpression of ADRB2 augmented TGF-β1-induced expression of various profibrotic proteins, including α-SMA, fibronectin, and N-cadherin, in comparison to the levels observed in control cells. Conversely, ADRB2 inhibition resulted in lower gene and protein expressions of α-SMA, Fibronectin, and N-cadherin in the TGF-β1-stimulated MRC-5 cells. These findings could indicate a significant involvement of ADRB2 in activating mesenchymal differentiation, resulting in heightened ECM deposition. However, the precise mechanism through which ADRB2 facilitates the differentiation of lung fibroblasts remains to be elucidated.

Accumulating evidence indicates that TGF-β1 promotes fibroblast-to-myofibroblast differentiation and ECM accumulation mainly through the SMAD-dependent canonical pathway, as well as through other noncanonical pathways [50, 51]. The canonical TGF-β1 signaling pathway requires binding to the TGF-β type II receptor (TGFBR2), followed by phosphorylation and activation TGF-β type I receptor (TGFBR1). Phosphorylated TGFBR1 then activates SMAD-dependent signaling and further activates or inhibits the transcription of TGF-β/Smad target genes [52]. Herein, we demonstrated that ADRB2 deficiency decreased TGF-β1-induced phos-SMAD2/3 expression in MRC-5 cells. In contrast, a gain of ADRB2 expression or activity further increased the expression of TGF-β1-induced phos-SMAD2/3 in MRC-5 cells. Both pharmacologic inhibition and knockdown of SMAD3 markedly blocked epinephrine-enhanced fibroblast differentiation in response to TGF-β1. Consistent with the findings in vitro, phos-Smad2/3 expression was also decreased by inhibition of ADRB2 in a bleomycin-induced mouse model. Collectively, these results indicate that ADRB2 regulates fibroblast differentiation *via* the TGFβ/Smad signaling pathway.

As a secondary messenger, ROS plays a vital role in myofibroblast differentiation by potentiating TGF-β1 signaling [17, 52, 53]. For example, in lung fibroblasts, DUOX1-derived H_2_O_2_ amplified the signaling output of the TGF-β1 pathway and exacerbated bleomycin-induced lung fibrosis [53]. Interestingly, ADRB2 has long been associated with ROS. The mechanisms of ROS production following receptor agonism and the signaling outcomes of such ROS are diverse in different cell types [54]. In this study, ADRB2-induced elevation of phosphorylated SMAD2/3 was associated with augmented ROS production and was blocked by pretreatment with the ROS scavenger NAC. This finding highlights a role for ADRB2 activation-derived ROS in signaling, thereby affecting the TGFβ/SMAD signaling.

TGFβ/SMAD signaling is regulated by various complex feedback loops at different levels [55]. In addition to dephosphorylation by phosphatases [56, 57], degradation of TGF-β1-induced SMAD by the ubiquitin-proteasome system has been recognized as a pivotal mechanism that ensures the termination of SMAD signaling [58]. By using TGF-β1-induced MRC-5 cells, we found that the addition of the broad-spectrum phosphatase inhibitor pervanadate had no effect on the loss of ICI-118,551-induced TGF-β1-mediated SMAD2/3 phosphorylation. Inactivation of the proteasome inhibits the protein degradation process and enhances homeostasis [59]. In contrast, pretreat with the proteasome inhibitor MG-132 markedly abrogated the downregulation of ICI-118,551-induced TGF-β1-dependent phos-SMAD2/3 expression and partially restored the decreases in TGF-β1-dependent fibrotic protein expression after ICI-118,551 treatment in MRC-5 cells. Based on this, we conclude that ADRB2 enhanced phos-SMAD2/3 expression at least partially through stabilization of the phos-SMAD2/3 protein. A CHX protein chase assay demonstrated that pharmacologic inhibition of ADRB2 reduced phos-SMAD2/3 in MRC-5 cells, while pharmacologic activation or overexpression of ADRB2 increased phos-SMAD2/3 protein stability in MRC-5 cells. And ADRB2 mediated K48-linked ubiquitination of phos-SMAD3 in an in vitro ubiquitination assay. The literature demonstrates that K48-linked ubiquitin chains constitute the predominant chain type, directing proteins toward degradation through proteasomal pathways [60]. This result further confirmed the role of ADRB2 in prolonging the duration of TGF-β1 signaling in fibroblasts by preventing phos-SMAD2/3 proteasome degradation.

Oxidative stress has been postulated as a primary driving factor in the progression of pulmonary fibrosis by interacting with multiple molecular mechanisms [17]. Studies have shown that N-acetyl cysteine, as a scavenger of ROS, blunts bleomycin-induced pulmonary fibrosis [53, 54, 61]. Our data demonstrate that elevated ROS induced by ADRB2 activation is crucial for driving fibroblast activation in MRC-5 cells. These observations led us to investigate whether ICI-118,551, in combination with NAC, exerts antifibrotic effects by modulating TGF-β1/Smad2/3 signaling. Interestingly, cotreatment with ICI-118,551 and NAC in bleomycin-challenged mice showed superior inhibition of fibrosis as compared to NAC or ICI-118,551 monotherapy.

Overall, we showed that ADRB2 was decreased in fibrotic lungs and TGF-β1-stimulated fibroblasts. Inhibition of ADRB2 attenuated bleomycin-induced pulmonary fibrosis in mice and reduced lung fibroblast-to-myofibroblast differentiation, proliferation, and migration by modulating TGF-β signaling activity through moderation of the stability of SMAD2/3, while NAC inhibited ADRB2 mediation of TGF-β signaling. ADRB2 is a novel regulator and target of pulmonary fibrosis. Adrb2 inhibition with ICI-118,551 can be a therapy for pulmonary fibrosis, while the combination of NAC with ICI-118,551 increased the efficacy, which might be a potential therapy for pulmonary fibrosis.

## Materials and methods

### Cell culture and treatment

The MRC-5 cell line and IMR-90 cell line were purchased from the American Type Culture Collection (CCL-171) and routinely cultured in DMEM with 10% (v/v) fetal bovine serum, 100 U/mL penicillin, and 100 mg/L streptomycin (Solarbio, Beijing, China) at 37 °C with 5% CO_2_. Both cell lines were tested and found to be free of mycoplasma.

ICI-118,551 hydrochloride (I127, Sigma-Aldrich), a highly selective inhibitor of ADRB2, and epinephrine hydrochloride (E4642, Sigma-Aldrich), an activator of ADRB2, were obtained from Sigma (Sigma-Aldrich; Merck KGaA, Darmstadt, Germany). These chemicals were prepared with H_2_O. The proteasomal inhibitor MG-132 (HY-13259, MedChemExpress), protein synthesis inhibitor cycloheximide (HY-12320, MedChemExpress), Smad3 selective inhibitor (E)-SIS3 (HY-13013, MedChemExpress), and ROS inhibitor N-acetylcysteine (NAC, HY-B0215, MedChemExpress) were dissolved in dimethylsulfoxide.

Upon reaching approximately 80% confluency, the cells were subjected to standard digestion using 0.25% trypsin/EDTA. A total of 1.0 × 10^5^ digested cells were seeded per well in 6-well plates (Corning Inc., Corning NY, USA). The next day, the cells were preincubated in the serum-deprived medium for 24 h and then incubated with different compounds (recombinant human TGFβ1 (rhTGF-β1), ICI-118,551, epinephrine).

### Plasmids and transfection

The ADRB2-overexpressing pIRES2-EGFP plasmid or the empty pIRES2-EGFP plasmid was synthesized and transfected into MRC-5 cells using Lipofectamine 3000 in accordance with the instructions provided by the manufacturer. Human SMAD3 shRNAs were designed and synthesized by Sangon Biotech and subsequently annealed and inserted into the pLKO.1 vector (Sangon, Shanghai, China).

### Cell counting Kit-8 (CCK8) assay

Cell viability was evaluated by a CCK8 assay according to the manufacturer’s instructions. In short, MRC-5 cells were seeded at a density of 3 × 10^3^ cells/well in 96-well microplates. Following incubation, the cells were treated with an inhibitor or agonist of ADRB2 for 24 h and 48 h. The cells were cultured with 10 μL of CCK-8 in each well at 37 °C for 2 h. Finally, the optical density was measured at 450 nm by a microplate reader.

### EdU assay

Cell proliferation was assessed using an EdU assay kit (RiboBio, Guangzhou, China). MRC-5 cells were seeded into 96-well plates at a density of 3 × 10^3^ cells per well. Following a 24-h treatment period, each well was exposed to 100 μL of 50 μM EdU medium for a 2-h duration at 37 °C. Subsequently, the cells were fixed in 4% paraformaldehyde for 30 min and permeabilized with 0.5% Triton X-100 for 10 min. After PBS rinses, the cells were subjected to a 30-minute incubation with 100 μL of 1× Apollo reaction cocktail. Then, 1× Hoechst 33342 was introduced and allowed to incubate for 30 minutes. Positive cells were visualized using fluorescence microscopy.

### Wound-healing assay

Cell migration was measured by wound-healing assays as described previously [62]. A marker pen was used to make a mark at the bottom of sterile 6-well plate. Then, MRC-5 cells were added to a sterile 6-well plate and cultured overnight. Cells were treated with an inhibitor or agonist of ADRB2 with or without TGF-β1. When the cells grew to 90–100% confluence, a sterile pipette tip was used to create a linear scratch across the cell surface, followed by PBS washing to remove cellular debris. The scratch was photographed using an inverted system microscope at 0 h, 24 h, and 48 h.

### Transwell assay

The treated MRC-5 cells were digested and resuspended in serum-deprived DMEM. A total of 100 μL of cell suspension (2 × 10^4^) was introduced into the upper transwell chamber, and 600 μL with complete culture medium was added to the lower chamber. The 24-well plate with a transwell chamber was incubated in a humidified incubator at 37 °C. After 24 h, invading cells were fixed with 4% paraformaldehyde for 30 min, then stained with crystal violet solution for 10 min, visualized using a microscope.

### Mouse model of bleomycin-induced pulmonary fibrosis and IPF

Eight-week-old C57BL/6 N male mice (6–8 weeks old) were obtained from Beijing Charles River Laboratory Animal Technology Co., Ltd. (Beijing, China) and maintained in a specific pathogen-free environment. All experiments involving animals were approved by the Institutional Animal Care and Use Committee. The mice were randomly divided into four groups. For bleomycin-induced pulmonary fibrosis, mice were anesthetized by isoflurane inhalation, and a dose of 1.5 U/kg bleomycin (Nippon Kayaku Co., Tokyo, Japan) dissolved in a total of 50 μL of saline was intratracheally administered once on Day 0. The control mice were administered 50 μL of saline in the same manner. For in vivo drug studies with an ADRB2 inhibitor, ICI-118,551 was diluted in saline, and mice were administered 2 mg/kg once daily by intraperitoneal injection with a total volume of 100 μL on Days 11–20 after bleomycin or saline treatment. N-acetyl cysteine (NAC), a scavenger of ROS, was diluted in saline, and mice were administered 500 mg/kg NAC daily by intragastric administration with a total volume of 100 μL on Days 11–20 after bleomycin treatment. Mice were sacrificed on Day 21. Then, the lung and BAL were harvested for histological studies, collagen determination, and biochemical analyses. The care and handling of animals adhered to the guidelines of the Henan Normal University Institutional Animal Care and Use Committee (IACUC, SMKX-2118BS1018), in accordance with the standards set by the Association of Animal Behavior and National Regulations.

IPF lung tissues and Control non-IPF lung tissue samples were recruited based on the ATS/ERS/JRS/ALAT Clinical Practice Guidelines at Henan Provincial Chest Hospital. IPF lung samples were obtained from patients undergoing open lung biopsy. Control lung tissues were from healthy lung tissue of other disease patients undergoing a surgical procedure. The research received approval from the Henan Provincial Chest Hospital Medical Research Ethics Committee (No. 2019-05-07), and informed consent was acquired from all patients before surgical procedures. The study was conducted in adherence to the principles outlined in The Code of Ethics of the World Medical Association (Declaration of Helsinki) pertaining to experiments involving human subjects.

### Isolation of mouse lung fibroblasts

Primary mouse lung fibroblasts were isolated from 2-month-old wild-type C57BL/6 mouse lung tissues by combining collagenase digestion and tissue adhere methods. Briefly, mouse lung tissues were cut into small pieces and digested by collagenase (1 mg/ml) in Hanks’ Balanced Salt Solution (HBSS) for 30 minutes. After centrifuged, cells were washed and resuspended in 10-cm dishes in DMEM (low glucose) containing 10% fetal bovine serum, 100 U/mL penicillin, and 100 mg/L streptomycin (Solarbio, Beijing, China) for 7 days. Confluent cells at P1 to P4 were used in the subsequent experiments.

### Quantitative real-time PCR (qRT‒PCR)

For transcriptional analysis of tissue samples or cultured cells, total RNA was extracted using RNeasy kits (Qiagen) as previously described [1]. RNA reverse transcription was executed utilizing Prime Script reverse transcriptase (Promega Corporation, Wisconsin, USA). Subsequently, qRT-PCR was carried out employing a Light Cycler 96 fluorescent quantitative PCR system (Roche) along with SYBR Premix Ex Taq (TaKaRa Biotechnology), utilizing cDNA as the template. The mean Ct values resulting from the triplicate analyses were normalized to the average Ct values of *ACTB*. The relative expression levels were computed using the formula 2^−ΔΔCt^. The primer pairs employed in this research are described in Table 1.

**Table 1:** Genes selected for expression analysis.

Primer name	Oligonucleotide sequence (5′-3′)
H-ACTB-F	5′-GGGAAATCGTGCGTGACAT-3′
H-ACTB-R	5′-CTCATTGCCAATGGTGATGA-3′
H-ACTA2-F	5′-CTCTGGACGCACAACTGGCATC-3′
H-ACTA2-R	5′-CACGCTCAGCAGTAGTAACGAAGG-3′
H-CDH2-F	5′-CGATAAGGATCAACCCCATACA-3′
H-CDH2-R	5′-TTCAAAGTCGATTGGTTTGACC-3′
H-FN1-F	5′-CGTGTACCATCGCAAACCG-3′
H-FN1-R	5′-ACCACATAGGAAGTCCCAGCA-3′
H-ADRB2-F	5′-GTGATCATGGTCTTCGTCTACT-3′
H-ADRB2-R	5′-CATGATGATGCCTAACGTCTTG-3′
M-Actb-F	5′-ATCGTGCGTGACATCAAAGA-3′
M-Actb-R	5′-CCACAGGATTCCATACCCAAG-3′
M-Collagen1-F	5′-GCTCCTCTTAGGGGCCACT-3′
M-Collagen1-R	5′-CCACGTCTCACCATTGGGG-3′
M-Adrb2-F	5′-TGCCCCTGGTGGTGATGGTCTTT-3′
M-Adrb2-R	5′-AAGCAGAACTTGGAGGACCTTCGG-3′

### Western blot (WB) analysis

Western blotting was performed as previously described [34]. Lung tissues or cells were homogenized and lysed with RIPA buffer (Beyotime, Shanghai, China) containing protease inhibitors (Beyotime). After centrifugation at 12,000×*g* for 15 min at 4 °C, the supernatants were collected and boiled at 95 °C for 10 min. The protein concentration was quantified by Pierce the BCA method. Subsequently, the acquired protein samples were subjected to separation via 8–12% SDS-PAGE and subsequently transferred onto PVDF membranes. After the samples were blocked with 5% BSA, the membranes were incubated with the designated primary antibodies overnight at 4 °C:: anti-ADRB2 (Thermo Fisher Scientific, #MA5-32570), anti-phos-ADRB2 (Thermo Fisher Scientific, #PA5-104767), anti-β-actin (Affinity, #T0022), anti-α-SMA (Cell Signaling Technology, #19245), anti-COL1A1 (Cell Signaling Technology, #72026), anti-N-cadherin (Cell Signaling Technology, #13116), anti-phos-SMAD2 (Cell Signaling Technology, #18338), anti-SMAD2 (Cell Signaling Technology, #5339), anti-phos-SMAD3 (Cell Signaling Technology, #9520), anti-SMAD3 (Cell Signaling Technology, #9523), anti-phos-TGFBR1 (Thermo Fisher Scientific, #PA5-40298) and anti-Fibronectin (Cell Signaling Technology, #26836). The membranes were then incubated with HRP-conjugated secondary antibody (Abcam, goat anti-rabbit, #ab205718 or goat anti-mouse, #ab6789) for 1 h at room temperature. Blots were visualized utilizing an enhanced chemiluminescence system (Bio-Rad, USA).

### Immunohistochemistry (IHC) and immunocytochemistry (ICC)

IHC was performed as previously described [1]. Briefly, lung tissues were first fixed using 4% paraformaldehyde, followed by a series of dehydration steps before being embedded in paraffin. Four-micrometer sections were deparaffinized and rehydrated, followed by antigen retrieval in citrate buffer (Beyotime) at 95 °C for 10 min. Then, the sections were blocked in endogenous peroxidase blocking solution (Beyotime) at 37 °C for 30 min and immunostained with primary antibodies (anti-ADRB2, anti-α-SMA, anti-phos-SMAD2/3) at 4 °C overnight. Subsequent to this, the lung sections underwent an incubation step with biotin-labeled secondary antibodies (Beyotime) at 37 °C for 30 min. The lung sections were subsequently subjected to development using a DAB working solution, followed by counterstaining with hematoxylin and eventual mounting using a mounting medium. Stained sections were photographed using light microscopy.

For ICC, cells were cultured on poly-l-lysine-coated coverslips, fixed using 4% paraformaldehyde for 30 min, and subsequently permeabilized with 0.03% Triton X-100 for 5 min. After three rinses with PBS, the cells were blocked with 5% goat serum in PBS for 30 min, followed by incubation with primary antibodies (anti-α-SMA, anti-SMAD2, anti-SMAD3) at 4 °C overnight. The cells were then stained with Alexa Fluor 488 (green)-conjugated secondary antibodies at 37 °C for 1 h. Finally, the nuclei were stained with DAPI. Stained cells were photographed by confocal microscopy (LSM 700, Zeiss, Jena, Germany).

### Hematoxylin and eosin (H&E) and Masson’s trichrome staining

H&E and Masson’s trichrome staining were performed as described [1]. The mouse lung tissues were fixed in 4% paraformaldehyde for 24 h and then dehydrated and embedded in paraffin. Four-micrometer sections were routinely deparaffinized in distilled water. H&E and Masson’s trichrome staining was performed for morphologic detection using a kit following the manufacturer’s instructions.

### Collagen gel contraction assay

Lung fibroblast contraction assay was carried out using a two-step cell contraction assay kit according to the manufacturer’s guidelines (CBA-201, Cell Biolabs, Inc, San Diego, CA). MRC-5 cells were suspended in fresh medium at a concentration of 3 × 10^6^ cells/ml. Then, the cell suspension was mixed with collagen stock solution at a 1:4 ratio of 250 μL/well in a 48-well dish, and incubated at 37 °C for 1 h. After collagen polymerization, 500 μL of medium with or without ICI-118,551/epinephrine/TGF-β1 was added to each well and further cultured at 37 °C for 48 h. After incubation, the collagen gels were then released and photographed at various time points. The gel contraction area was measured using ImageJ software (ImageJ 1.52q). The gel contraction rate was shown as a contracted area that accounted for the initial gel release surface area.

### Detection of ROS

The intracellular ROS level was assessed using a ROS assay kit (Applygen Technologies Inc., Beijing, China). Briefly, the cells were incubated with 10 µM dihydroethidium at 37 °C for 30 min in the dark. Following incubation, the cells were washed twice with PBS, and the fluorescence intensity was analyzed by microplate reader (excitation at 535 nm; emission at 610 nm).

### In vitro ubiquitination assay

HA-SMAD3, together with Myc-Ub plasmids, were transfected into MRC-5 cells. They were first treated with 20 μM ICI-118,551/Epinephrine before harvesting. 48 h later, cells were treated with 20 μM MG132 for 4 h, stimulated with 10 ng/ml TGF-β1 for 30 min, and then lysed. After the addition of 1% SDS, the cell lysates were subjected to boiling for 10 min. Then, the cell lysates were diluted to a concentration of 0.1% SDS using lysis buffer and subjected to immunoprecipitation using anti-HA beads. The immunoprecipitated samples were subjected to immunoblot analysis employing an anti-Myc antibody.

### Micro-CT imaging

Twenty-one days after bleomycin administration, in vivo micro-CT analysis of the whole lung was performed. Briefly, mice were lightly anesthetized with isoflurane and fixed in the supine position. Micro-CT images were acquired using a Bruker SkyScan 1276 micro-CT (Bruker, Kontich, Belgium). The scanning parameters were as follows: 60 kV X-ray tube voltage and 200 µA anode current; Cu filter of 0.5 mm, resulting in a total acquisition time of approximately 10 min. The reconstructed images were superposed by Insta-Recon software (Bruker microCT, Kontich, Belgium).

### Measurement of hydroxyproline

Total collagen in the right lung was tested using a hydroxyproline assay kit as previously described [63, 64]. In short, the total right lung tissue was homogenized in 10 times the volume of distilled water and hydrolyzed at 120 °C for 3 h after the addition of an equal volume of 12 M hydrochloric acid. After brief cooling, the mixture was centrifuged at 10,000×*g* for 3 min. Ten microliters of the supernatant were transferred into a 96-well plate and then evaporated at 60 °C until dry. Following this, 100 μL of the Chloramine T/Oxidation Buffer Mixture was introduced into each well, and the plate was left to incubate at room temperature for 5 min. Subsequently, 100 μL of the diluted DMAB reagent was added to the samples, which were then incubated at 60 °C for 90 min. The absorbance of each sample was read at 560 nm, and the data were calculated as µg hydroxyproline per right lung.

### Statistical analyses

Data were statistically analyzed using GraphPad Prism 7 (GraphPad Software, Inc., San Diego, CA, USA). The normal distribution was assessed using the Shapiro–Wilk test. For non-normally distributed sample data, comparisons between two groups were analyzed using the Mann–Whitney *U* test, whereas, for normally distributed data, an unpaired Student’s *t*-test was employed. All presented data are expressed as the mean ± standard deviation (SD), with statistical significance considered at a *P* value of less than 0.05.

### Supplementary information

Supplemental material

unedited gel

## Data Availability

All data generated or analyzed during this study are included in this published article.
