# Supplementary material for: ADRB2 inhibition combined with antioxidant treatment alleviates lung fibrosis by attenuating TGFβ/SMAD signaling in lung fibroblasts
Source: Cell Death Discov. 2023 Nov 4;9:407. doi: 10.1038/s41420-023-01702-9 (PMC10624856; doi:10.1038/s41420-023-01702-9)
Supplement: Supplementary file 1 — Supplemental material [file 41420_2023_1702_MOESM1_ESM.docx]

**Supplementary Figure 1**


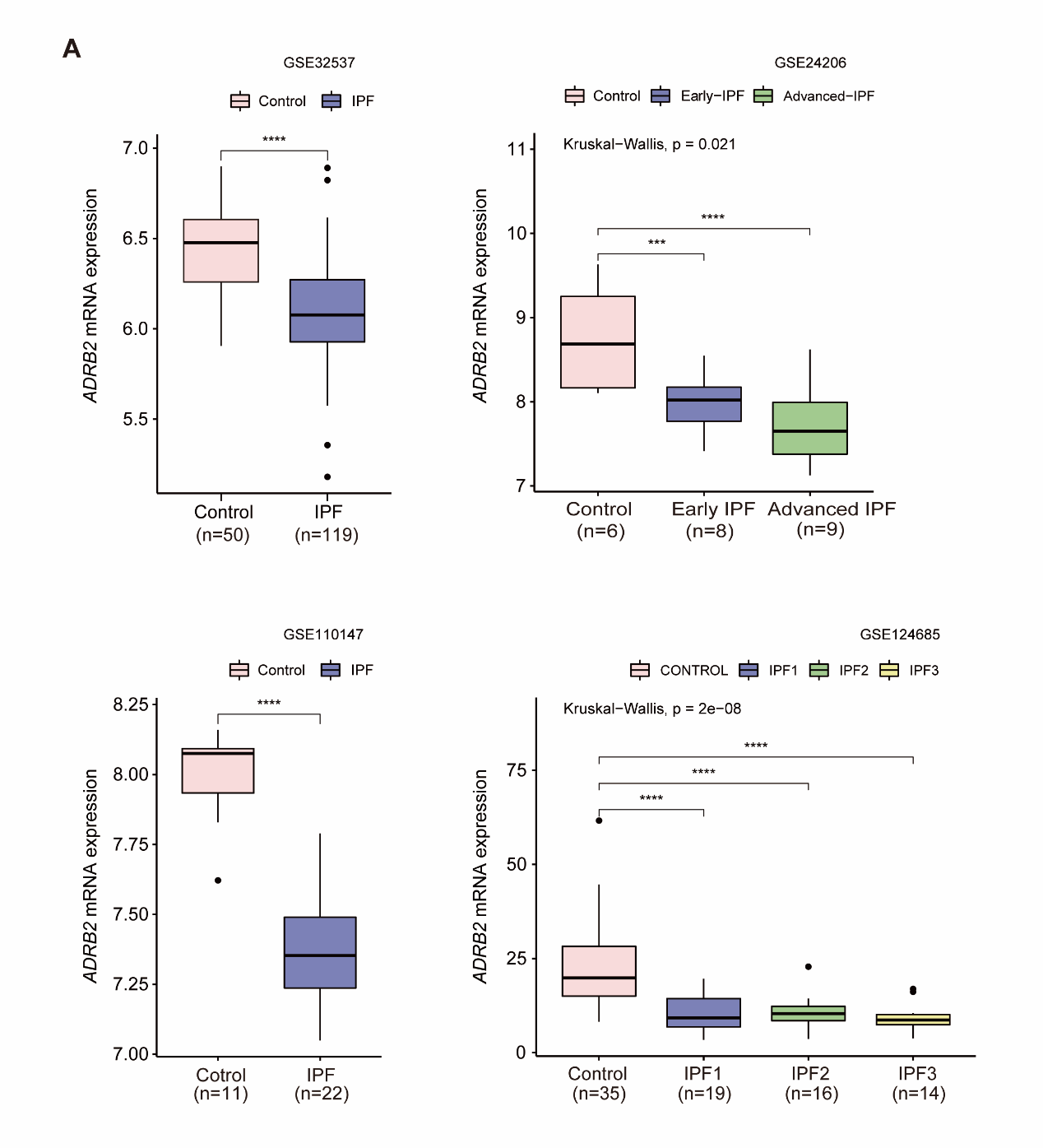


*ADRB2* mRNA expression was down-regulated in lungs of IPF patients. Analysis of publicly available microarray data (accession number GSE32537, GSE24206, GSE110147, GSE124685) in the lung samples from IPF patients and control subjects. Data are presented as Box-plots. Unpaired t test and variance analysis with Holm’s adjustment for independent samples. * adjusted-P value < 0.05; ** adjusted-P value < 0.01; *** adjusted-P value < 0.001; **** adjusted-P value < 0.0001.

**Supplementary Figure 2**


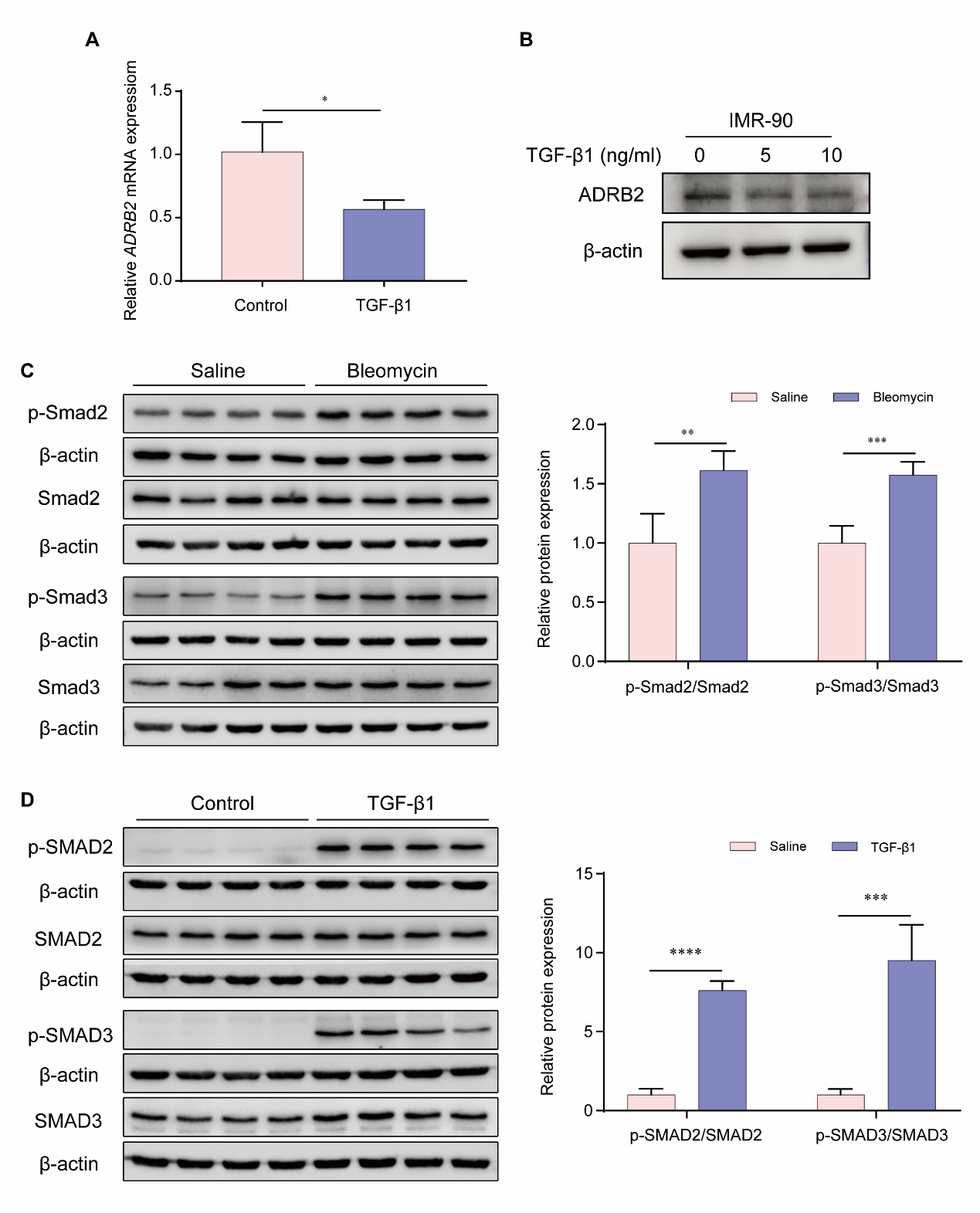


The expression of ADRB2, p-SMAD2/3 and SMAD2/3 in bleomycin-treated fibrotic mouse lung tissues and TGF-β1-stimulated lung fibroblasts. The mRNA (A) and protein (B) expression of ADRB2 in control- and TGF-β1-induced IMR-90 cells by qRT-PCR and WB analysis, respectively (n = 3). (C) WB analysis of bleomycin-induced p-Smad2/3 and Smad2/3 expression in mouse lung tissues (n = 4). (D) WB analysis of TGF-β1-induced p-SMAD2/3 and SMAD2/3 expression in MRC-5 cells (n = 4). *P＜0.05; **＜0.01; ***P＜0.001; ****P＜0.001.

**Supplementary Figure 3**


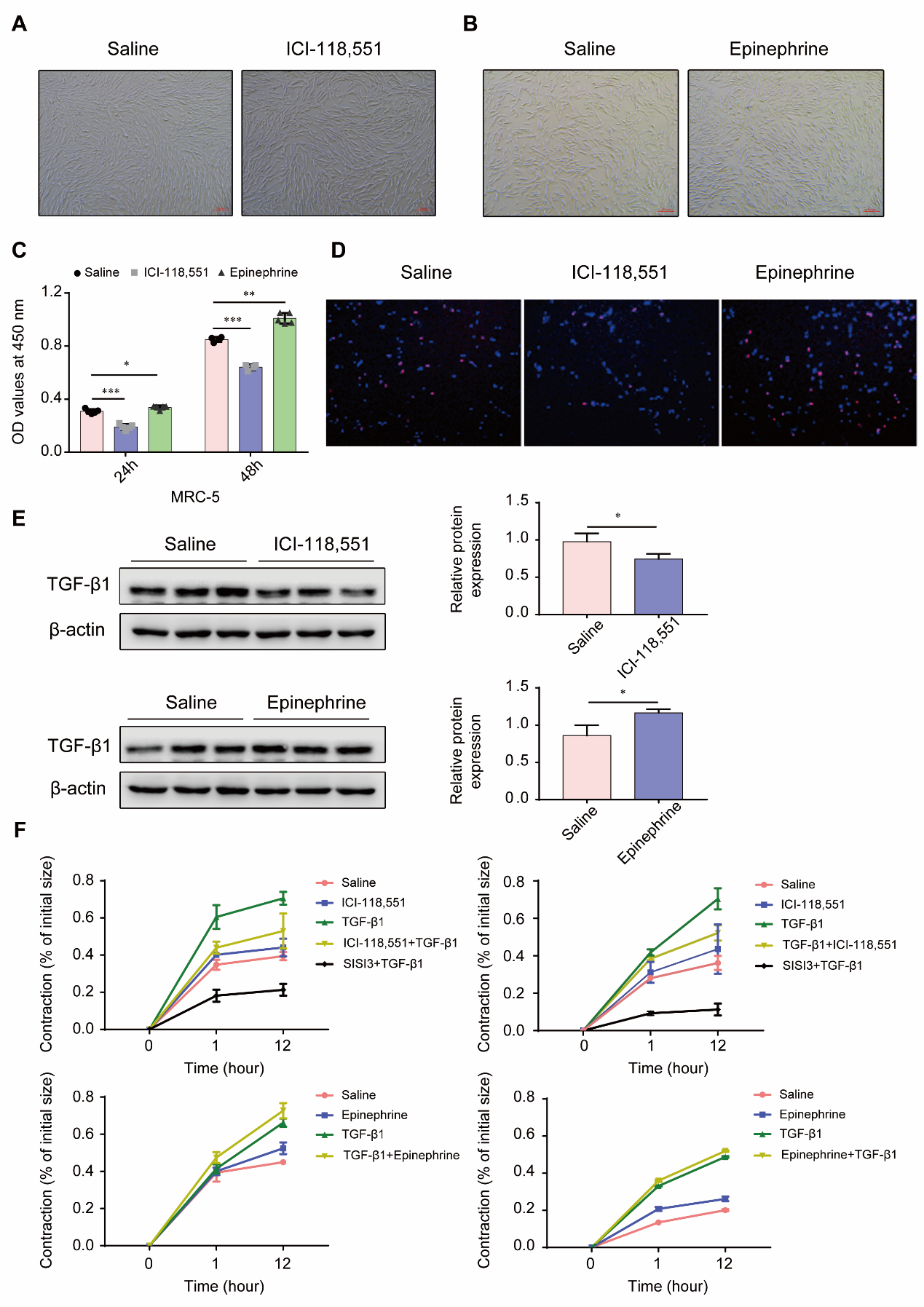


ADRB2 affected the viability, proliferation, and activation of fibroblasts. (A-B) Phase contrast microscopy images showed morphology of 20 μM ICI-118,551 or 20 μM epinephrine treated MRC-5 cells (n = 3). (C-E) The cell viability (n = 3) (C), cell proliferation (n = 3) (D) and the protein expression of TGF-β1 (n = 3) (E) in MRC-5 cells were treated with 20 μM ICI-118,551 or 20 μM epinephrine. (F) Quantification of % gel constriction relative to the initial area of MRC-5 cells was presented after release. Data are shown as the mean± SD. *P＜0.05; **＜0.01; ***P＜0.001.

**Supplementary Figure 4**


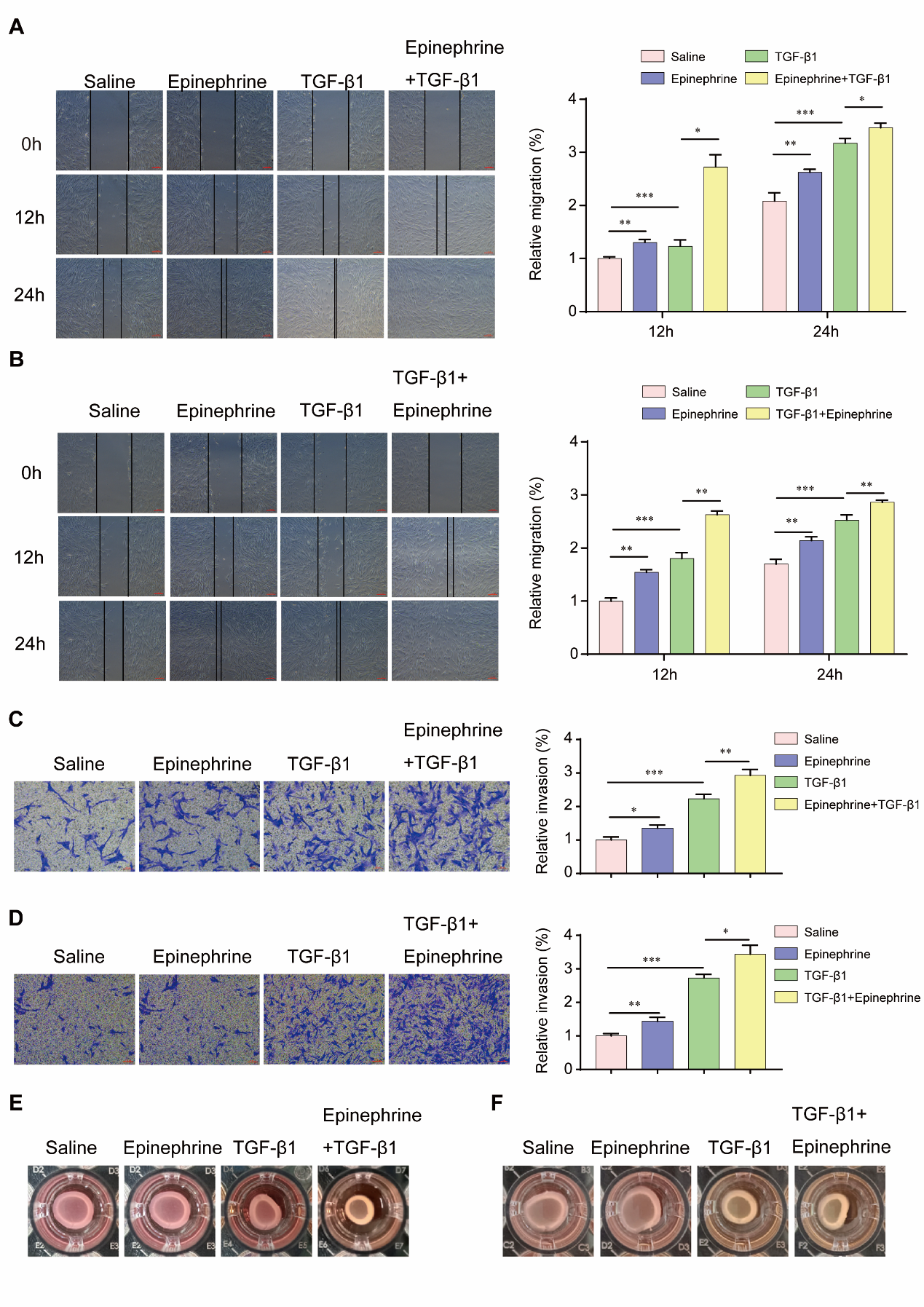


Activation of ADRB2 was able to augment migration, and activation of fibroblasts in with TGF-β1 induction or not. (A-B) The migration of MRC-5 cells in different treatment groups was tested by the scratch wound assay. Cells were treated with 20 μM epinephrine for 24 h, then stimulated with TGF-β1 (10 ng/mL) for 24 h (A, n = 3). Cells were treated with 10 ng/mL TGF-β1 for 24 h, then stimulated with 20 μM epinephrine for 24 h (B, n = 3). (C-D) Transwell assay was used to detect the invasion ability of MRC-5 cells. Cells were treated with 20 μM epinephrine for 24 h, prior to challenge with TGF-β1 (10 ng/mL) (C, n = 3). Cells were treated with 10 ng/mL TGF-β1 for 24 h, then stimulated with 20 μM epinephrine for 24 h (D, n = 3)ok. (E-F) Collagen contraction assay was used to evaluate the myofibroblast activation in different treatment groups. Cells were treated with 20 μM epinephrine for 24 h, then stimulated with TGF-β1 (10 ng/mL) for 24 h (E, n = 3). Cells were treated with 10 ng/mL TGF-β1 for 24 h, then stimulated with 20 μM epinephrine for 24 h (F, n = 3). Data are shown as the mean± SD. *P＜0.05; **＜0.01, ***P＜0.001.

**Supplementary Figure 5**


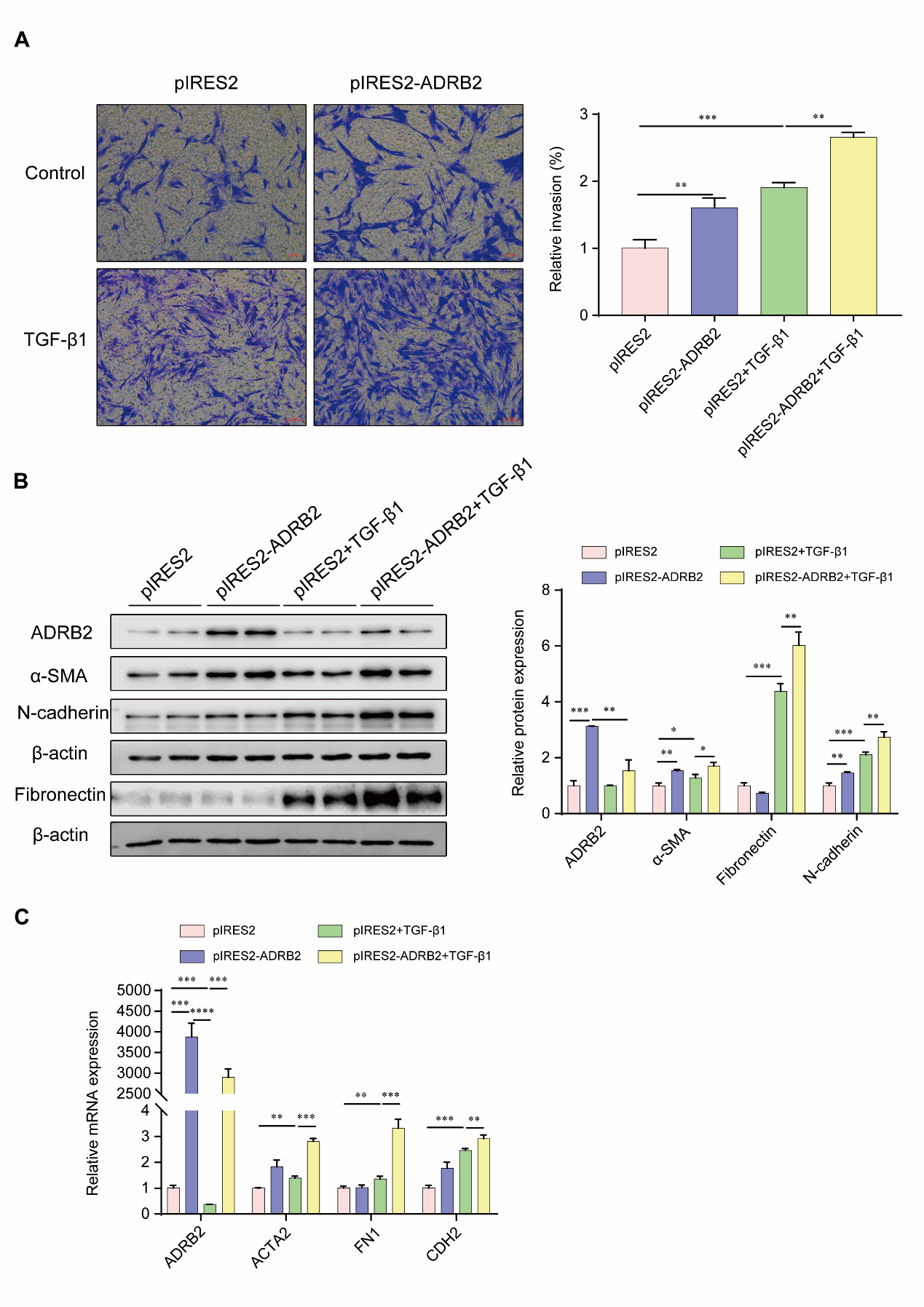


Overexpression of ADRB2 aggravated TGF-β1-induced myofibroblast migration and differentiation in MRC-5 cells. (A) Transwell assay was used to detect the invasion ability of MRC-5 cells. Cells were transfected with ADRB2 plasmid or empty control plasmid for 24 h, prior to challenge with TGF-β1 (10 ng/mL) (n = 3). The protein (B) and transcript (C) expression levels of ADRB2, α-SMA, Fibronectin and N-cadherin in MRC-5 cells were overexpression of ADRB2 for 24 h, prior to challenge with TGF-β1 (10 ng/mL) (n = 3). Data are shown as the mean± SD. *P＜0.05; **＜0.01, ***P＜0.001.

**Supplementary Figure 6**


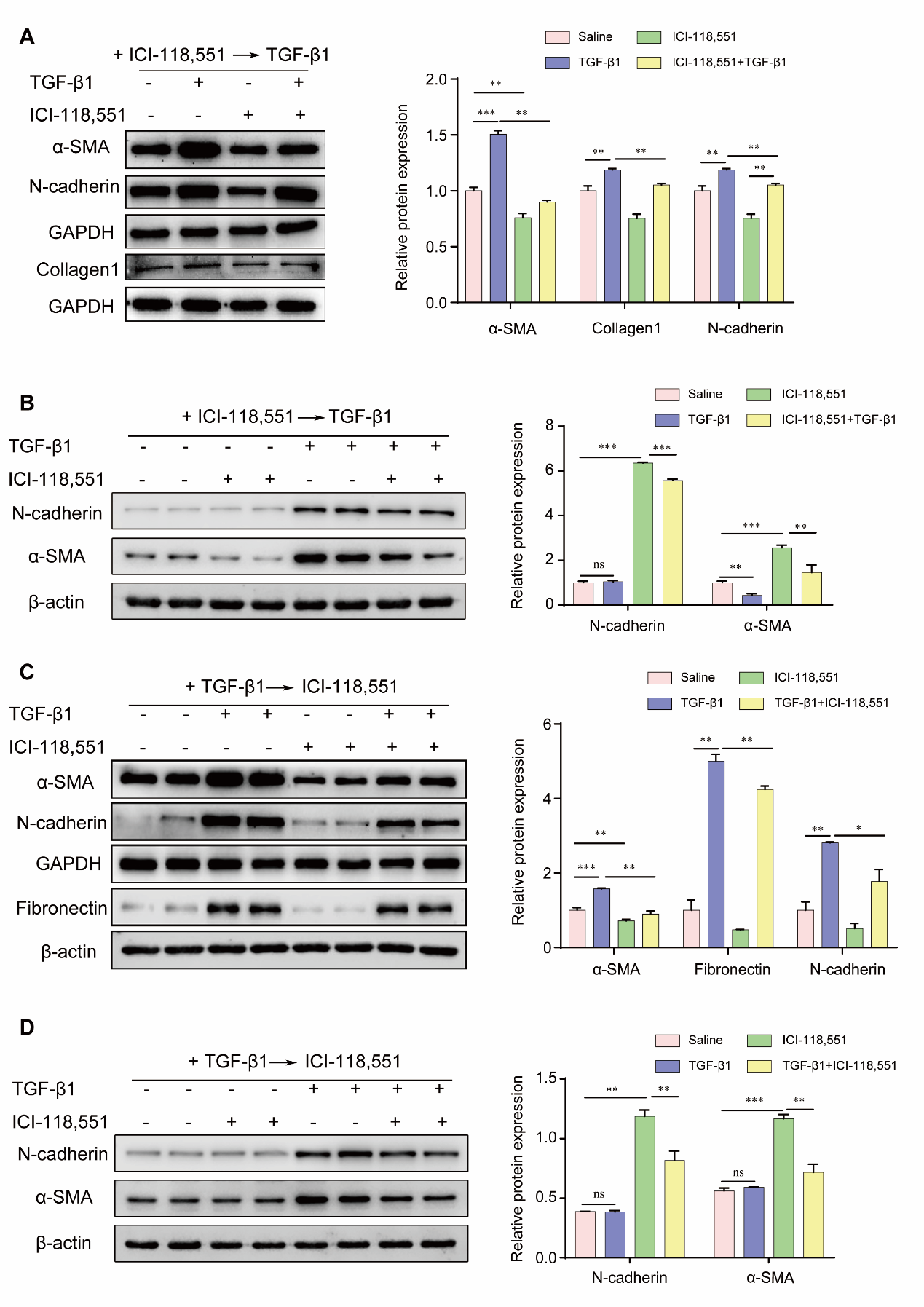


ADRB2 inhibition prevented and reversed TGF-β1-induced IMR-90 and primary mouse lung fibroblasts cells differentiation. (A) Effects of 24 h pre-treatment of IMR-90 cells with 20 μM ICI-118,551 (prevention protocol) on TGF-β1-induced expression of α-SMA, N-cadherin and Collagen1 (n = 3). (B) Effects of 24 h pre-treatment of primary mouse lung fibroblasts cells with 20 μM ICI-118,551 on TGF-β1-induced expression of N-cadherin and α-SMA (n = 3). (C) WB analysis of α-SMA, Fibronectin and N-cadherin expression in TGF-β1-generated myofibroblasts treated with 20 μM ICI-118,551 for 24 h (reversal protocol) (n = 3). (D) Protein expression levels of N-cadherin and α-SMA in primary mouse lung fibroblasts cells stimulated with TGF-β1 for 24 h, prior to treat with 20 μM ICI-118,551 (n = 3). Data are shown as the mean± SD. *P＜0.05; **＜0.01, ***P＜0.001.

**Supplementary Figure 7**


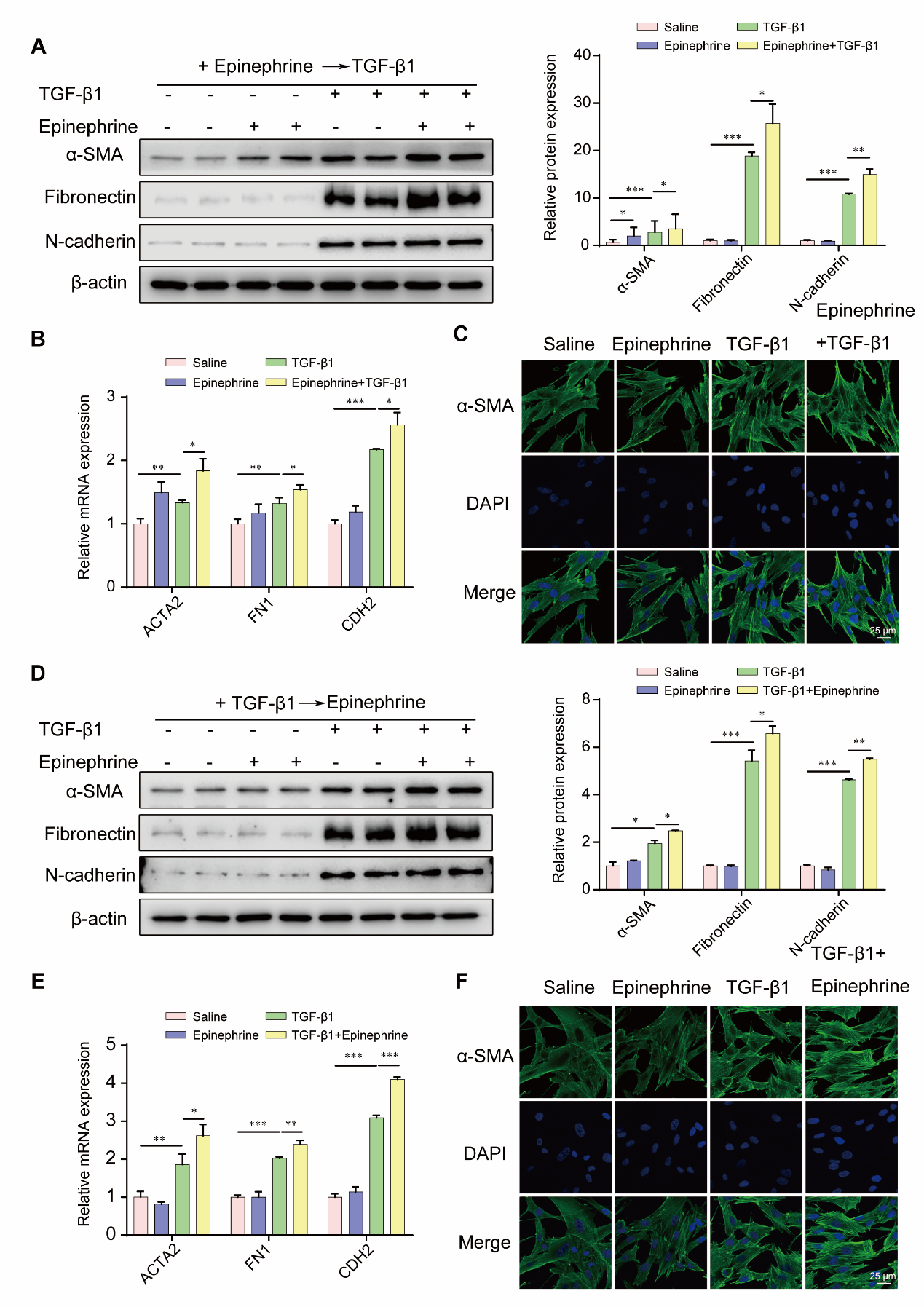


ADRB2 was sufficient to induce fibroblast activation and ECM production. (A) Protein and (B) transcript expression levels of α-SMA, fibronectin and N-cadherin in MRC-5 cells were treated with 20 μM epinephrine for 24 h, prior to challenge with TGF-β1 (10 ng/mL) (n = 3). (C) Representative immunofluorescence staining of α-SMA expression in MRC-5 cells were treated with 20 μM epinephrine for 24 h, prior to challenge with TGF-β1 (10 ng/mL) (n = 3). (D) Western blot and (E) qRT-PCR analysis of α-SMA, Fibronectin and N-cadherin expression in TGF-β1-generated myofibroblasts treated with 20 μM epinephrine for 24 h (n = 3). (F) Immunofluorescence analysis of α-SMA in TGF-β1-generated myofibroblasts treated with 20 μM epinephrine for 24 h. Data are shown as the mean± SD. *P＜0.05; **P＜0.01, ***P＜0.001.

**Supplementary Figure 8**


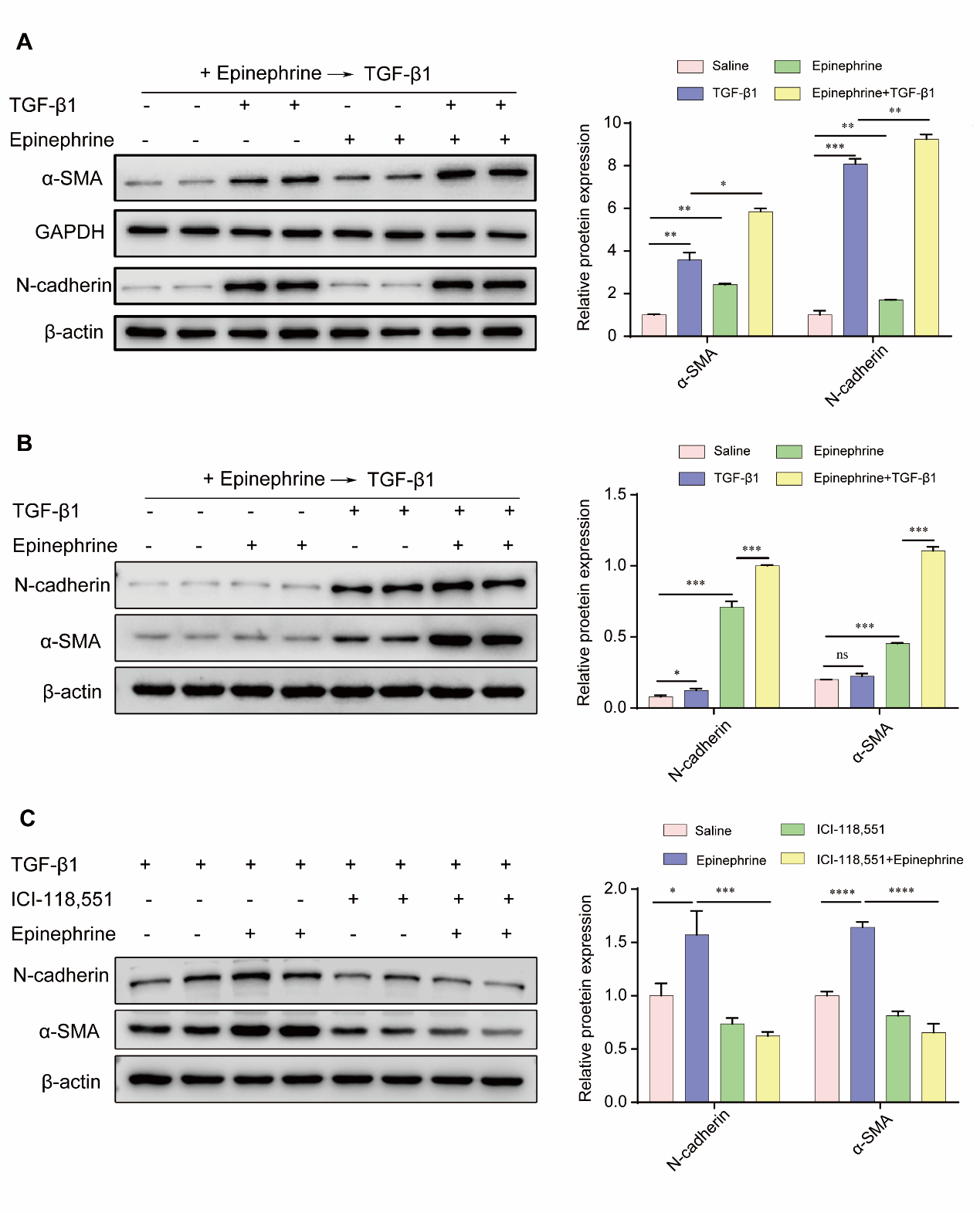


ADRB2 activation aggravated TGF-β1-induced fibroblast-to-myofibroblast differentiation. Effects of 24 h pretreatment of IMR-90 cells (A) and primary mouse lung fibroblasts cells (B) with 20 μM epinephrine on TGF-β1–induced protein expression of α-SMA and N-cadherin. (C) MRC-5 cells were treated with 20 μM epinephrine for 24 h in the presence or absence 20 μM ICI-118,551, then cells were treated with TGF-β1 (10 ng/mL) for 24 h. The protein expression of α-SMA and N-cadherin was determined by WB analysis (n = 3). Data are shown as the mean± SD. *P＜0.05; **P＜0.01; ***P＜0.001; ****P＜0.0001.

**Supplementary Figure 9**


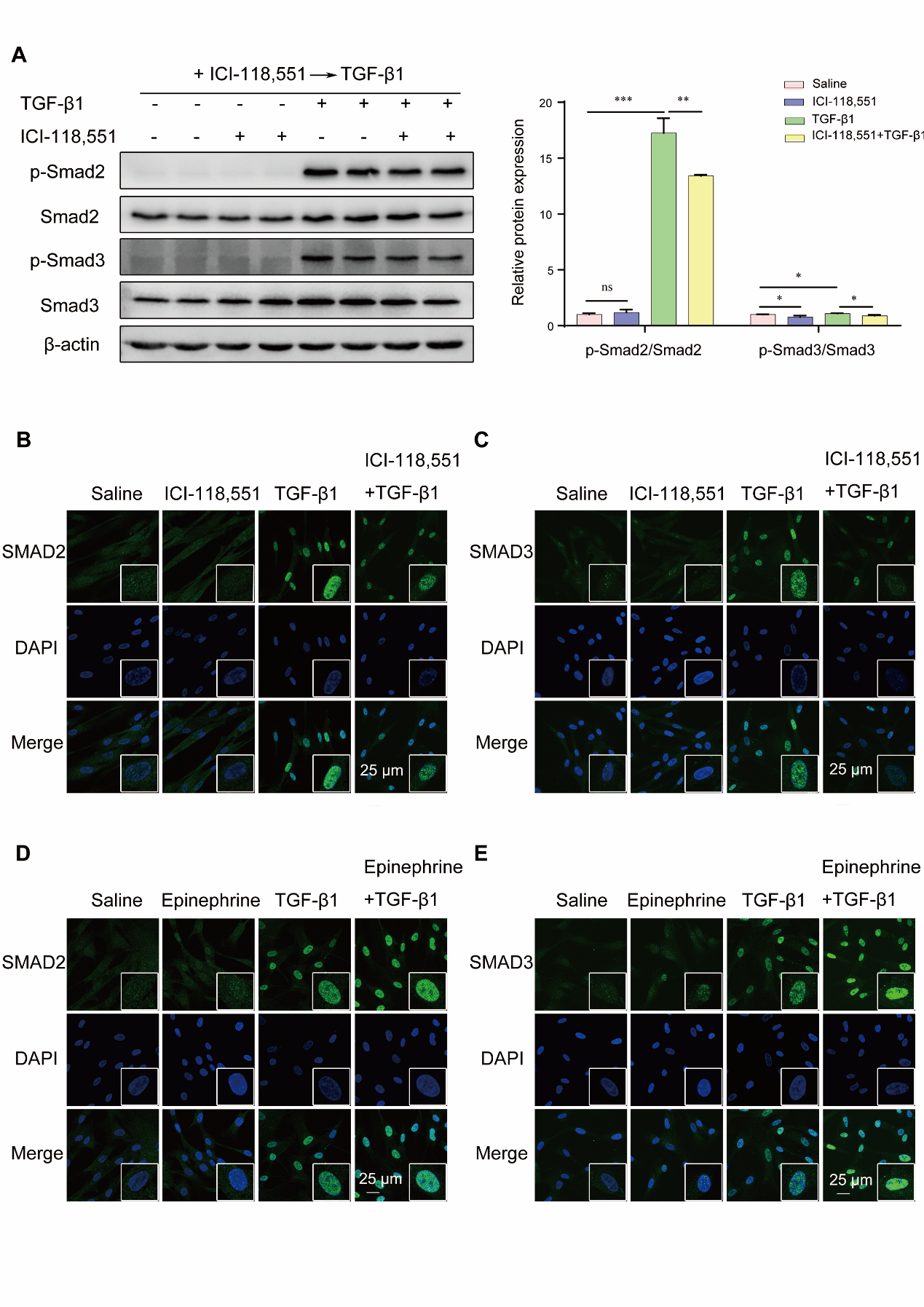


Activation of ADRB2 inhibited, while inhibition of ADRB2 promoted, SMAD2/3 nuclear export. (A) WB analysis of Smad2, Smad3 expression and their phosphorylated forms in primary mouse lung fibroblasts cells treated with or without 20 μM ICI-118,551 for 24 h, prior to challenge with TGF-β1 (10 ng/mL). (B-C) Representative immunofluorescence staining of SMAD2 (B) and SMAD3 (C) expression in MRC-5 cells with or without 20 μM ICI-118,551 for 24 h, prior to challenge with TGF-β1 (10 ng/mL). (D-E) Immunofluorescence microscopic analysis of SMAD2 (D) and SMAD3 (E) expression in MRC-5 cells with or without 20 μM epinephrine for 24 h, prior to challenge with TGF-β1 (10 ng/mL).

**Supplementary Figure 10**


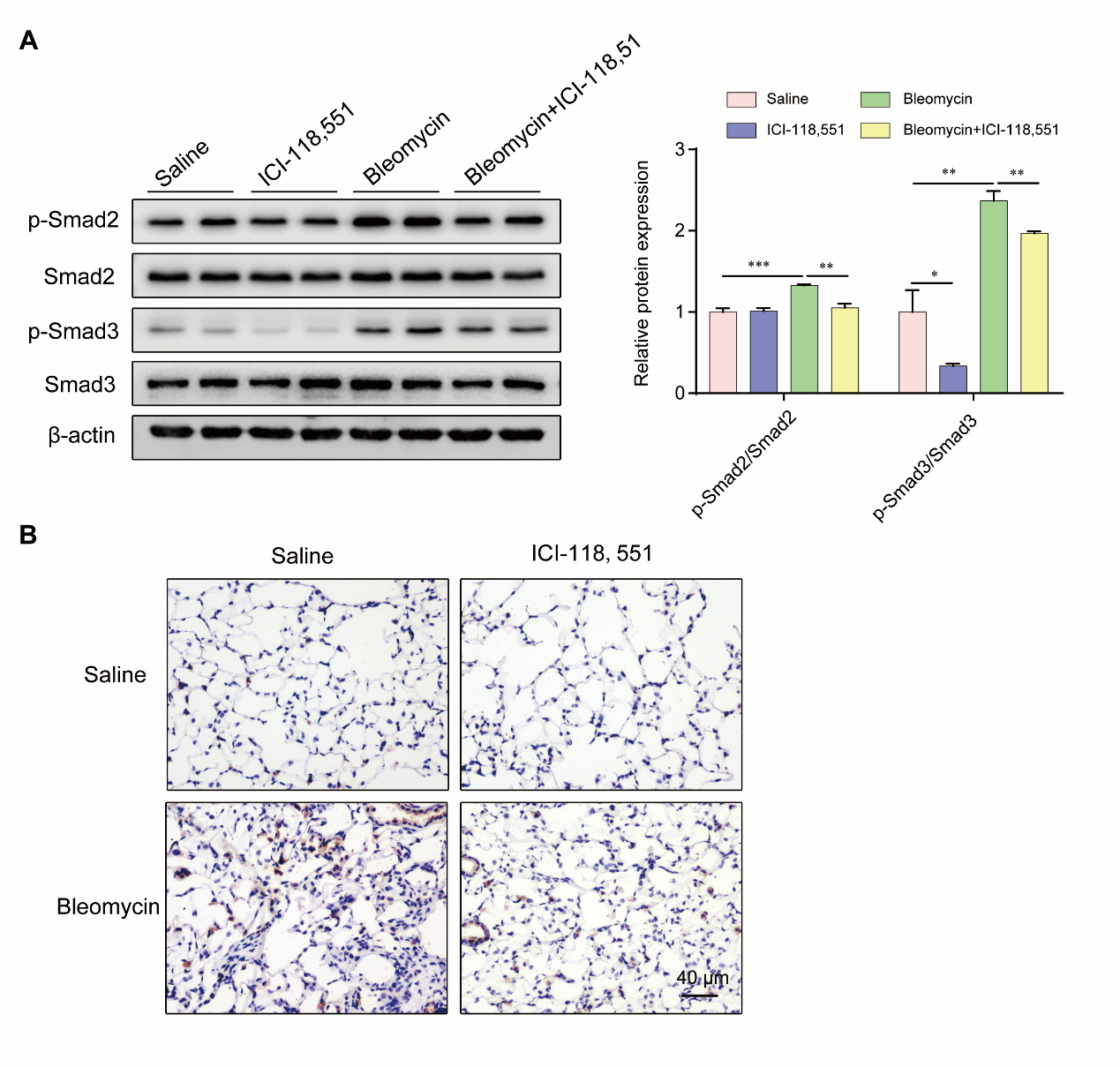


Decreased phos-Smad2/3 expression in bleomycin-induced fibrotic mice lungs. (A) Smad2/3 and phos-Smad2/3 protein expression in lung sections from saline- and ICI-118,551-treated following sham procedure or bleomycin treatment by WB analysis (n = 3). (B) Representative IHC staining of phos-Smad2/3 of lung sections from saline- and ICI-118,551-treated mice after intratracheal bleomycin or saline instillation (n = 3). Data are shown as the mean± SD. *P＜0.05; ***P＜0.001.

**Supplementary Figure 11**


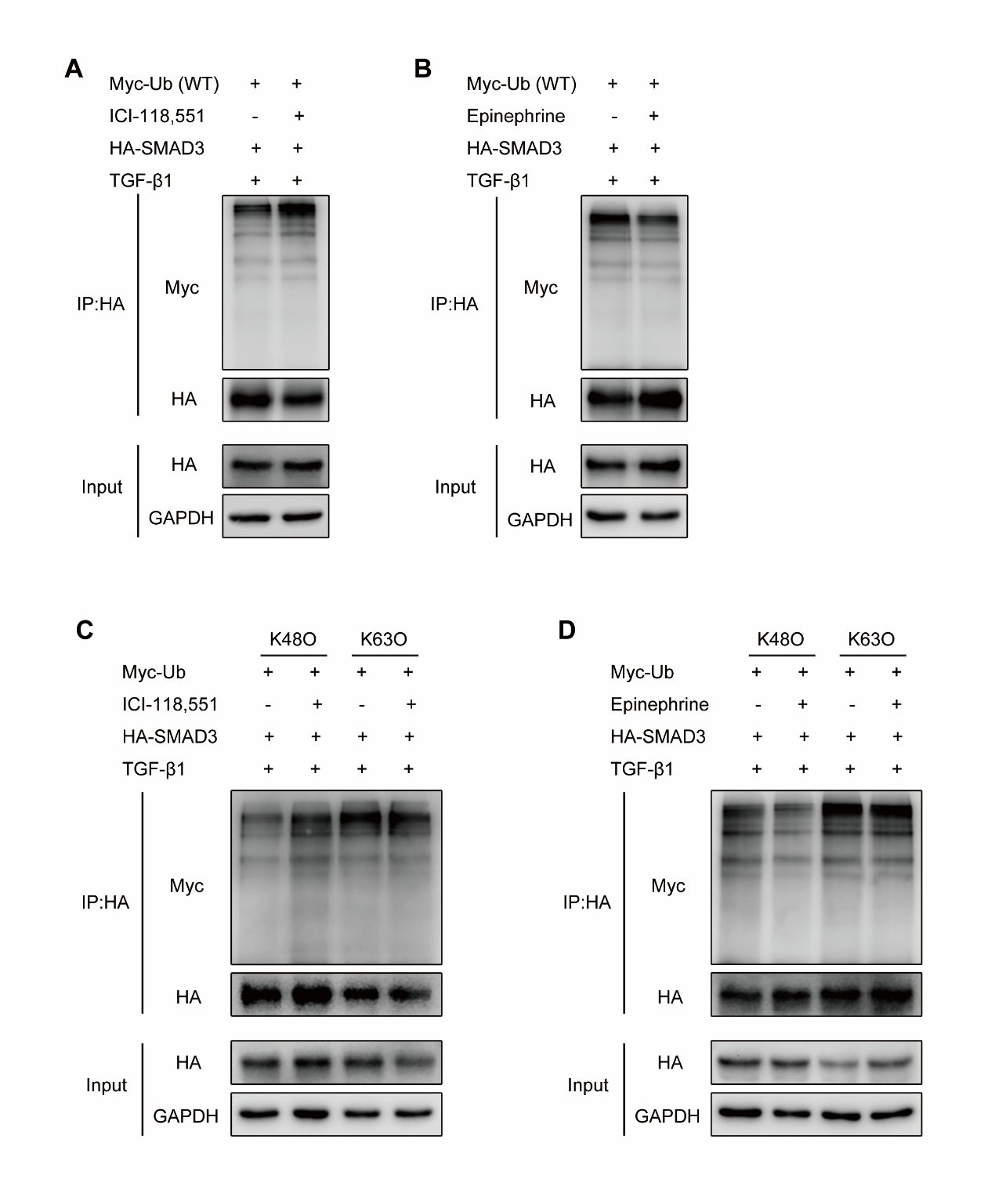


ADRB2 mediated K48-linked ubiquitination of phos-SMAD3. MRC-5 cells were transfected with the expression vectors of HA-SMAD3 and WT Myc-Ub or its mutants as indicated and then treated with 20 μM ICI-118,551 (A, C) /Epinephrine (B, D) for 48 h. The cells were pre-treated with 20 μM MG132 for 4 h, followed by TGF-β1 (10 ng/ml) treatment for 30 min before lysis. The cell lysates were subjected to ubiquitination assay with anti-HA beads. Immuno-complexes and lysates (input) were analyzed by Western blotting with the indicated antibodies. K48O and K63O mutants replace lysine residues with arginine residues at all sites except the specified one.
