# Supplementary material for: ADRB2 inhibition combined with antioxidant treatment alleviates lung fibrosis by attenuating TGFβ/SMAD signaling in lung fibroblasts
Source: Cell Death Discov. 2023 Nov 4;9:407. doi: 10.1038/s41420-023-01702-9 (PMC10624856; doi:10.1038/s41420-023-01702-9)

Full unedited gel for Figure 1E.

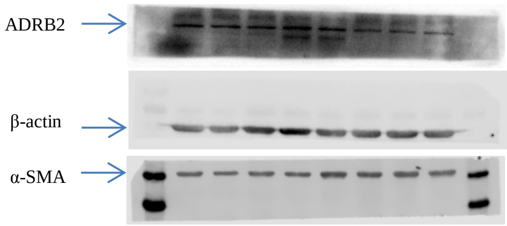

Full unedited gel for Figure 1G.

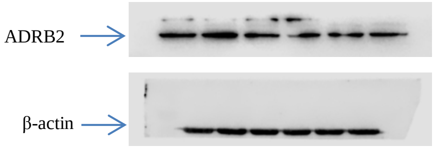

Full unedited gel for Figure 2C.

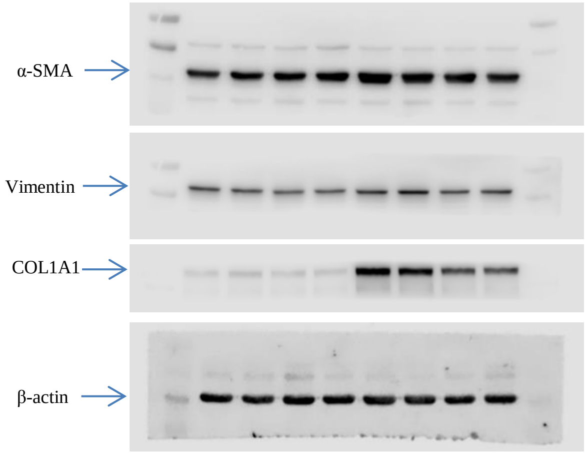

Full unedited gel for Figure 4A.

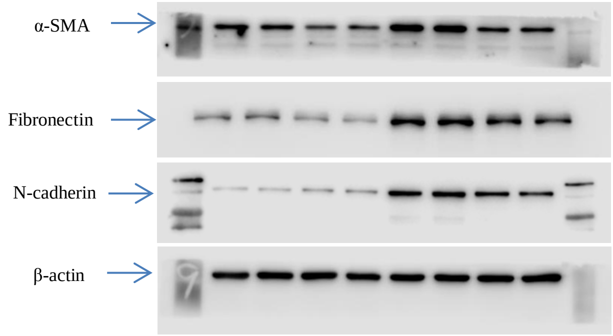

Full unedited gel for Figure 4D.

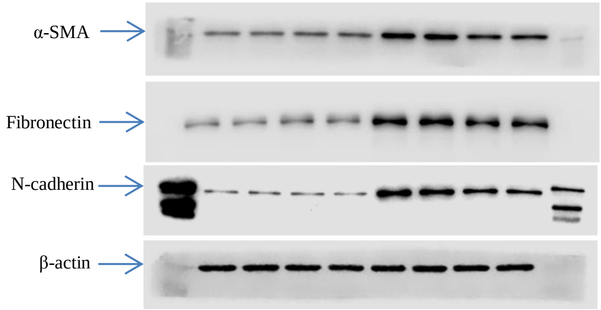

Full unedited gel for Figure 5A.

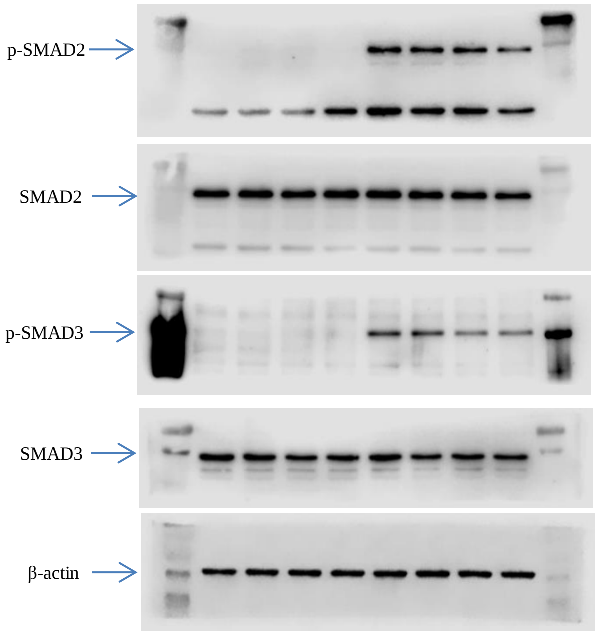

Full unedited gel for Figure 5B.

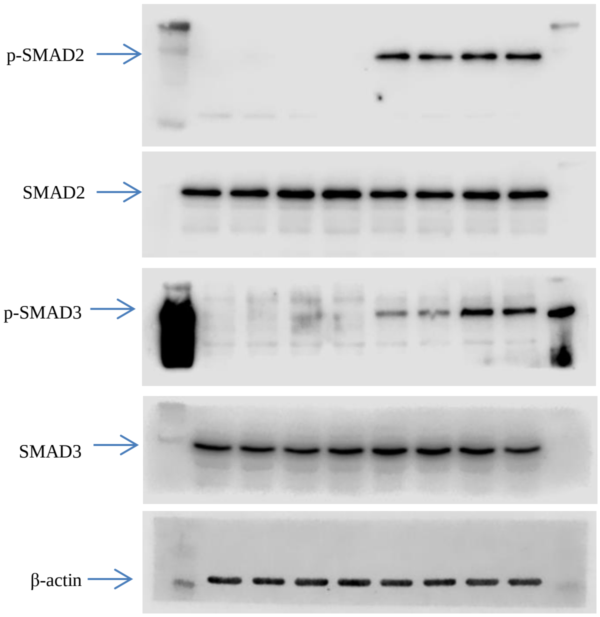

Full unedited gel for Figure 5C.

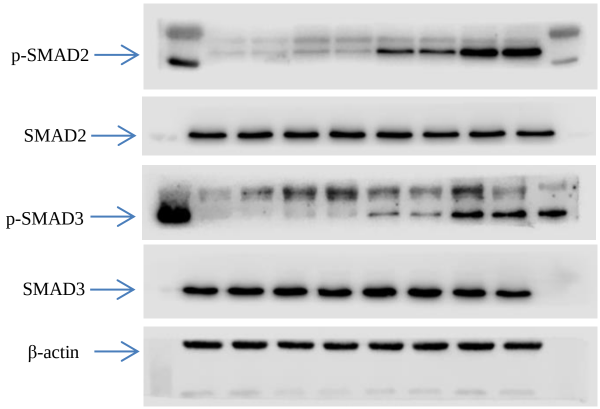

Full unedited gel for Figure 5D.

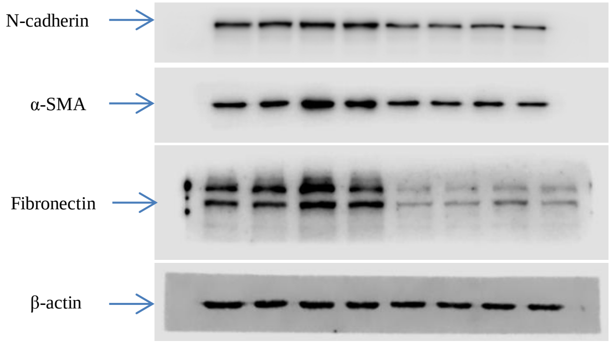

Full unedited gel for Figure 5E.

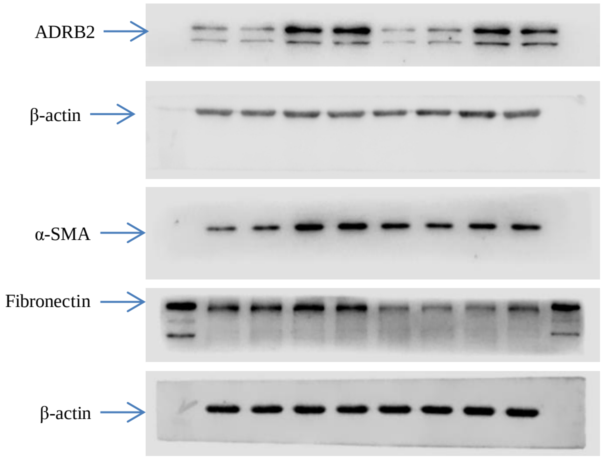

Full unedited gel for Figure 5F.

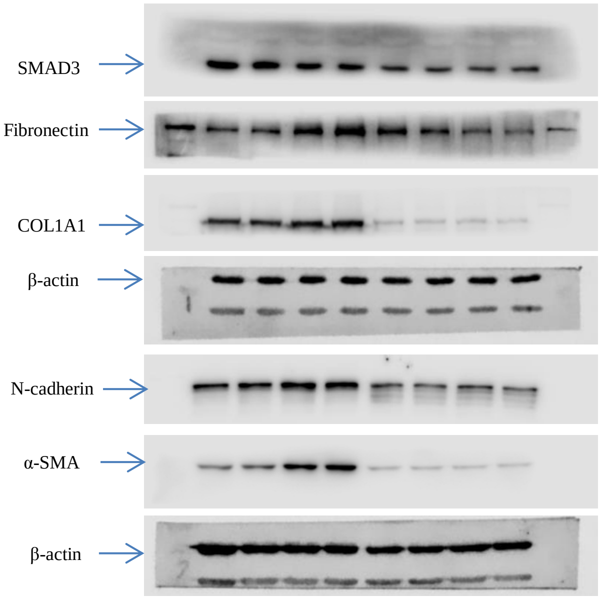

Full unedited gel for Figure 6B.

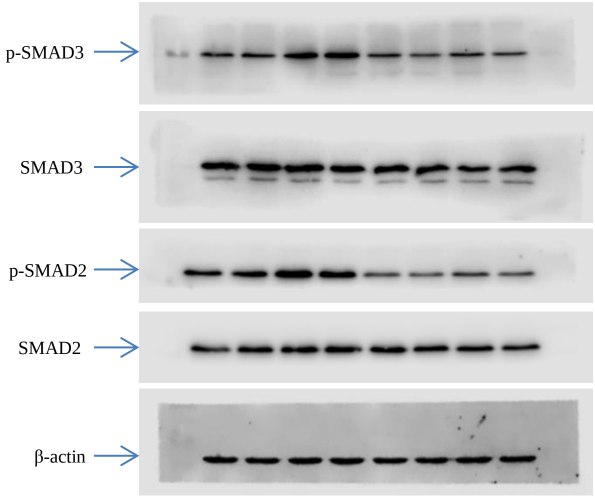

Full unedited gel for Figure 6C.

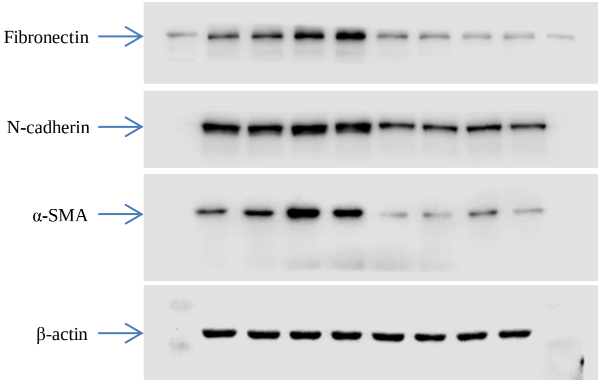

Full unedited gel for Figure 7A.

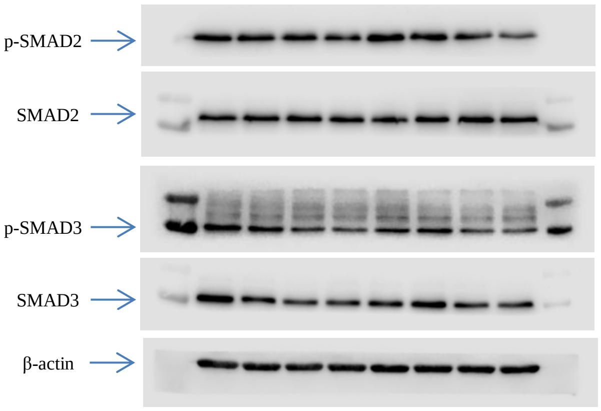

Full unedited gel for Figure 7B.

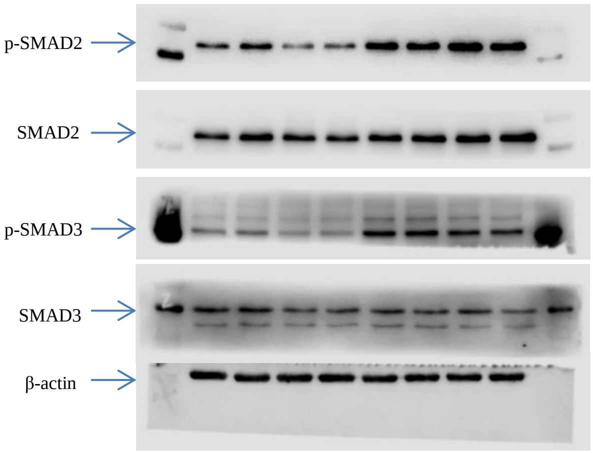

Full unedited gel for Figure 7C.

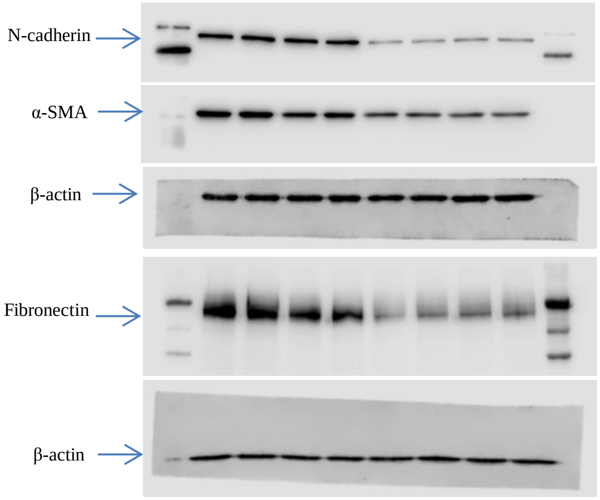

Full unedited gel for Figure 7D.

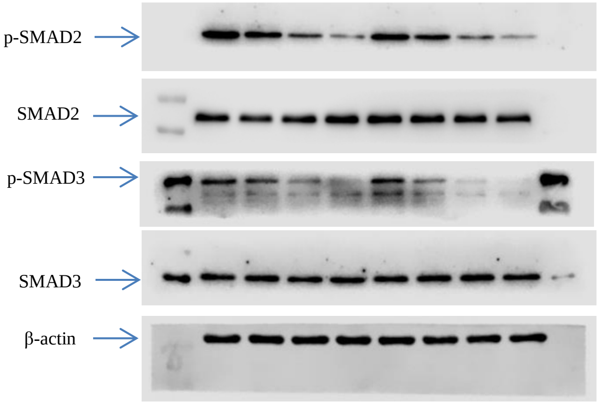

Full unedited gel for Figure 7E.

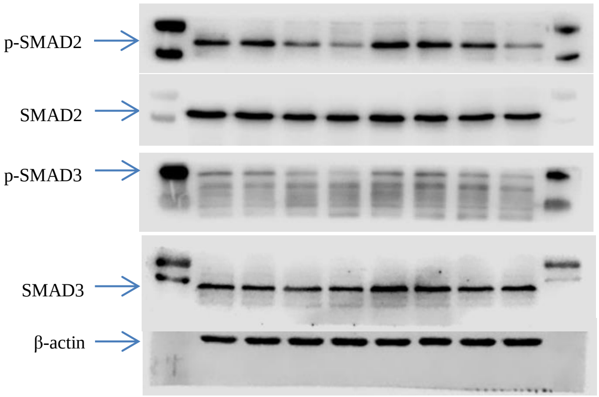

Full unedited gel for Figure 8C.

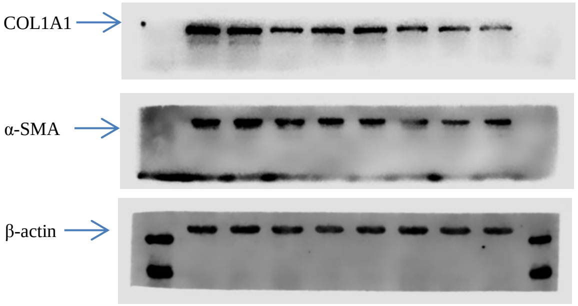

Full unedited gel for Supplementary Figure 2B.

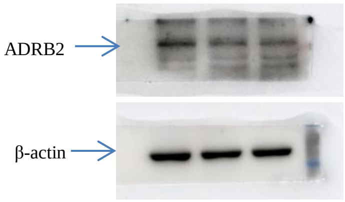

Full unedited gel for Supplementary Figure 2C.

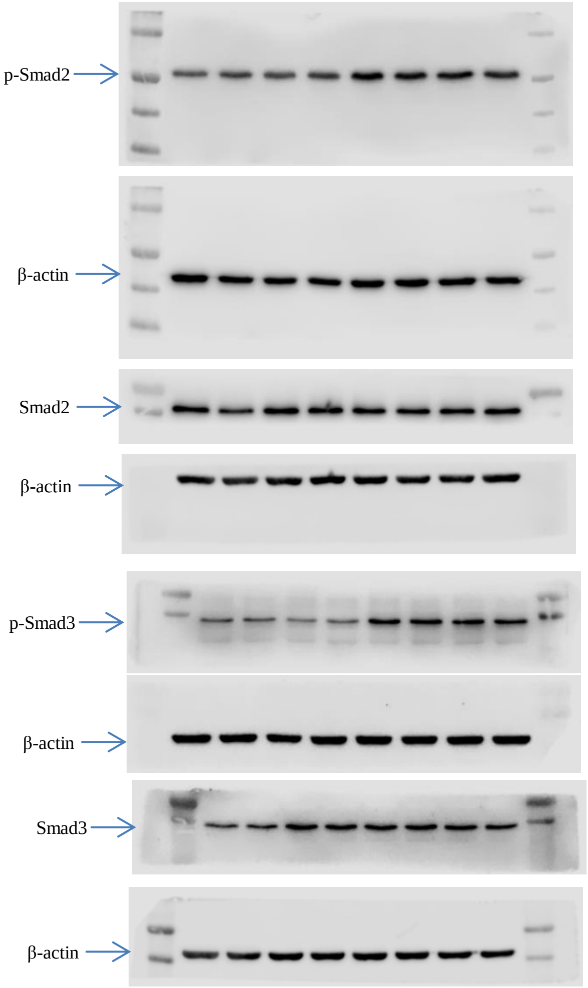

Full unedited gel for Supplementary Figure 2D.

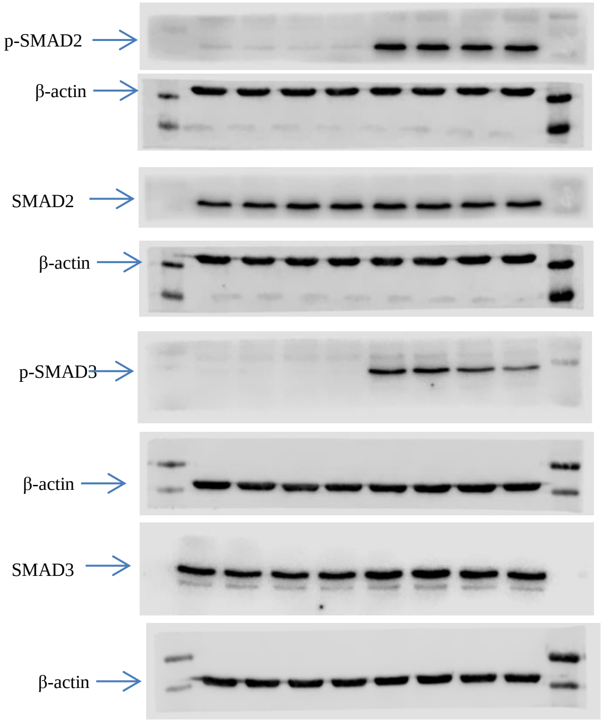

Full unedited gel for Supplementary Figure 3E.

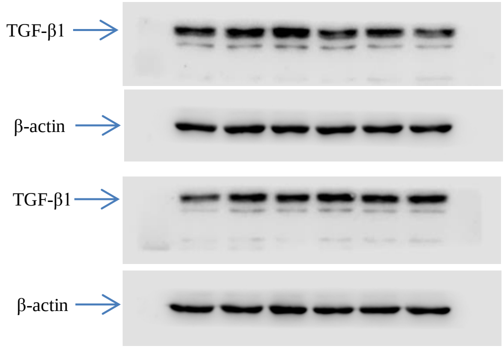

Full unedited gel for Supplementary Figure 5B.

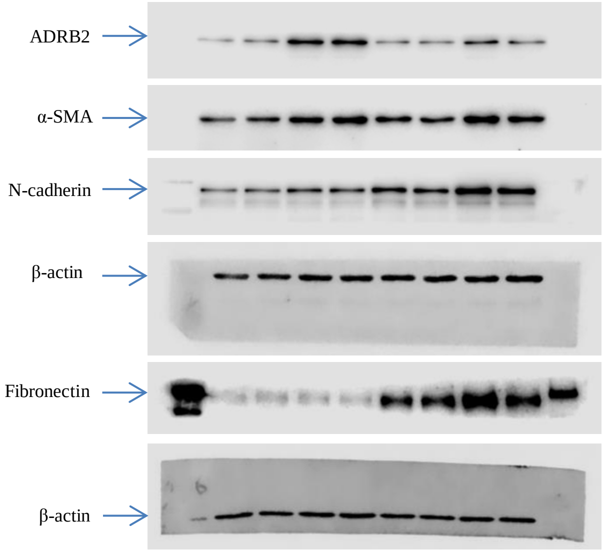

Full unedited gel for Supplementary Figure 6A.

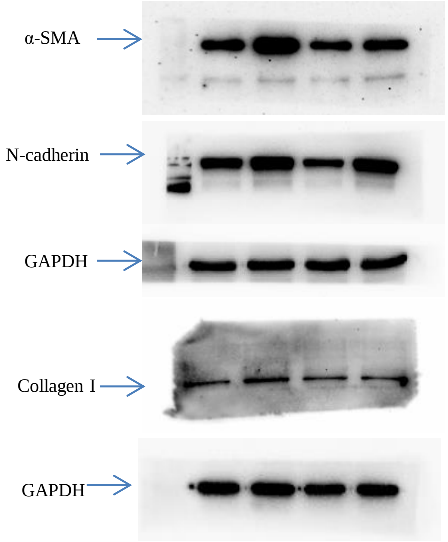

Full unedited gel for Supplementary Figure 6B.

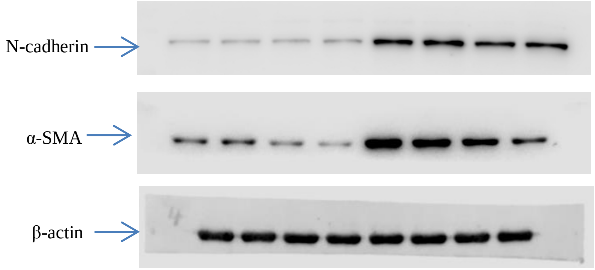

Full unedited gel for Supplementary Figure 6C.

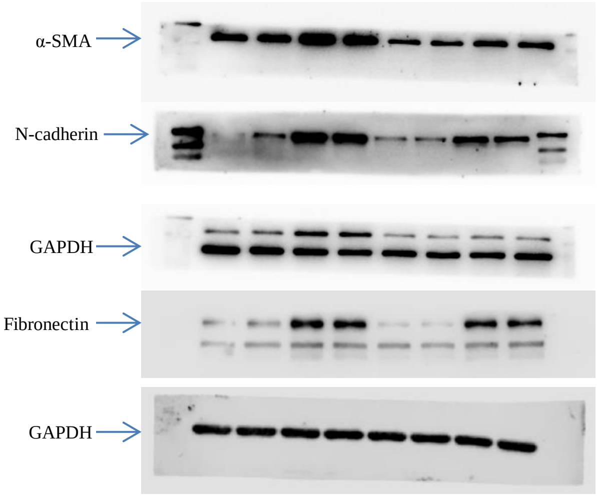

Full unedited gel for Supplementary Figure 6D.

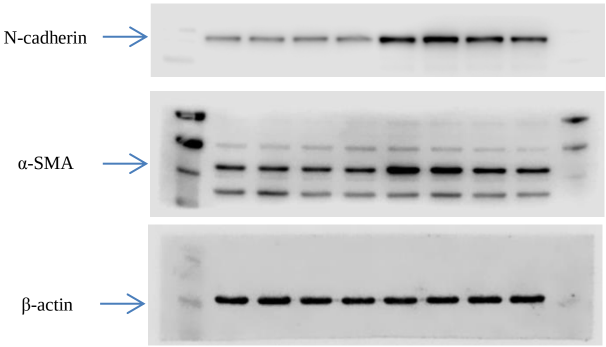

Full unedited gel for Supplementary Figure 7A.

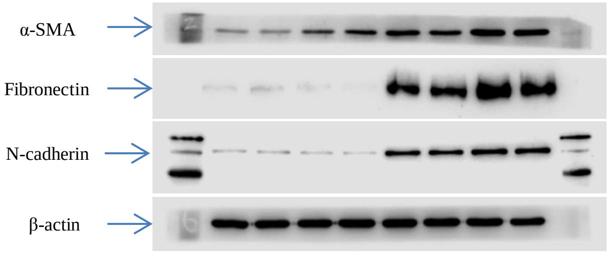

Full unedited gel for Supplementary Figure 7D.

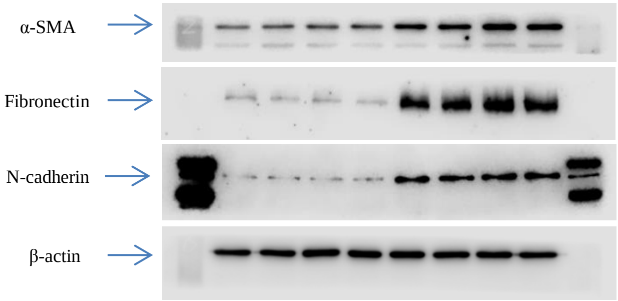

Full unedited gel for Supplementary Figure 8A.

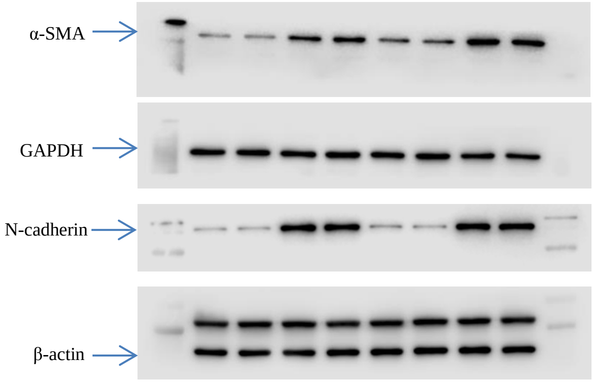

Full unedited gel for Supplementary Figure 8B.

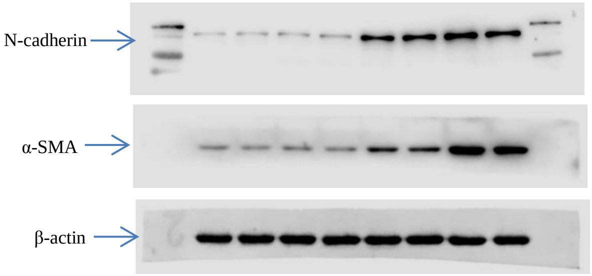

Full unedited gel for Supplementary Figure 8C.

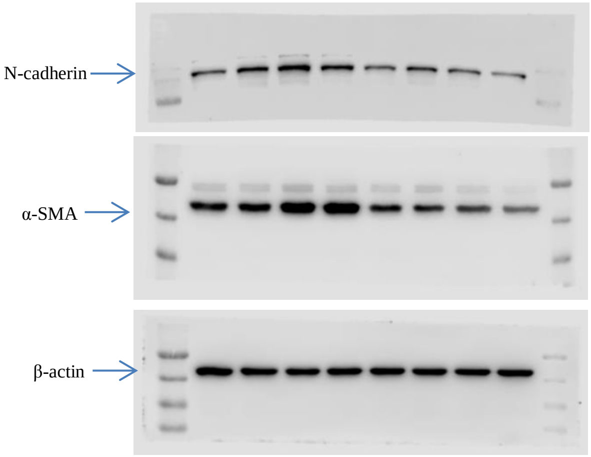

Full unedited gel for Supplementary Figure 9A.

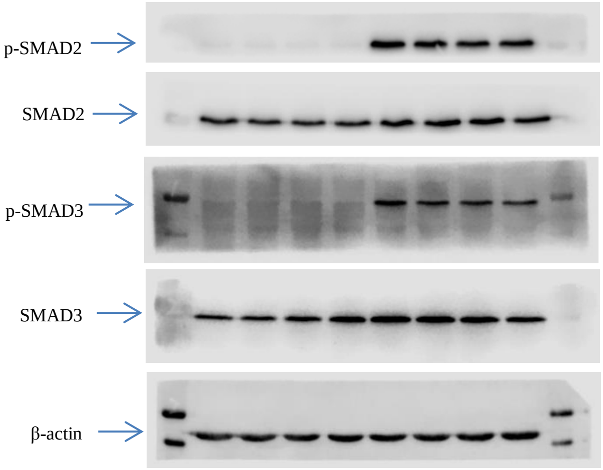

Full unedited gel for Supplementary Figure 10A.

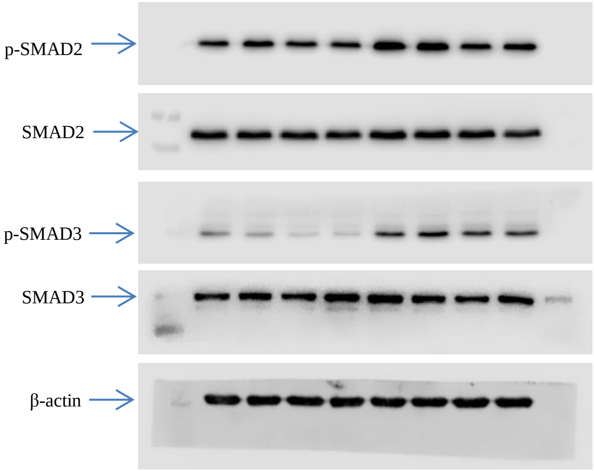

Full unedited gel for Supplementary Figure 11A.

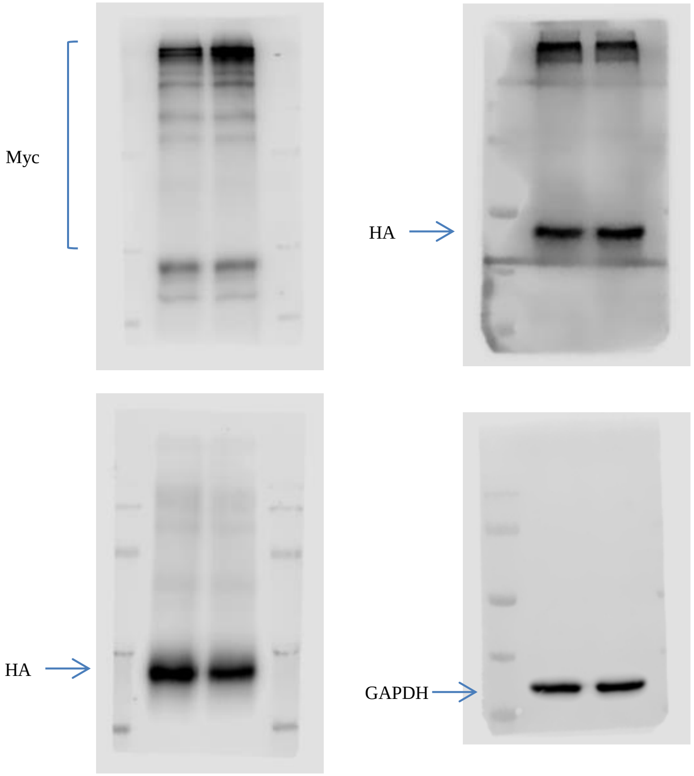

Full unedited gel for Supplementary Figure 11B.

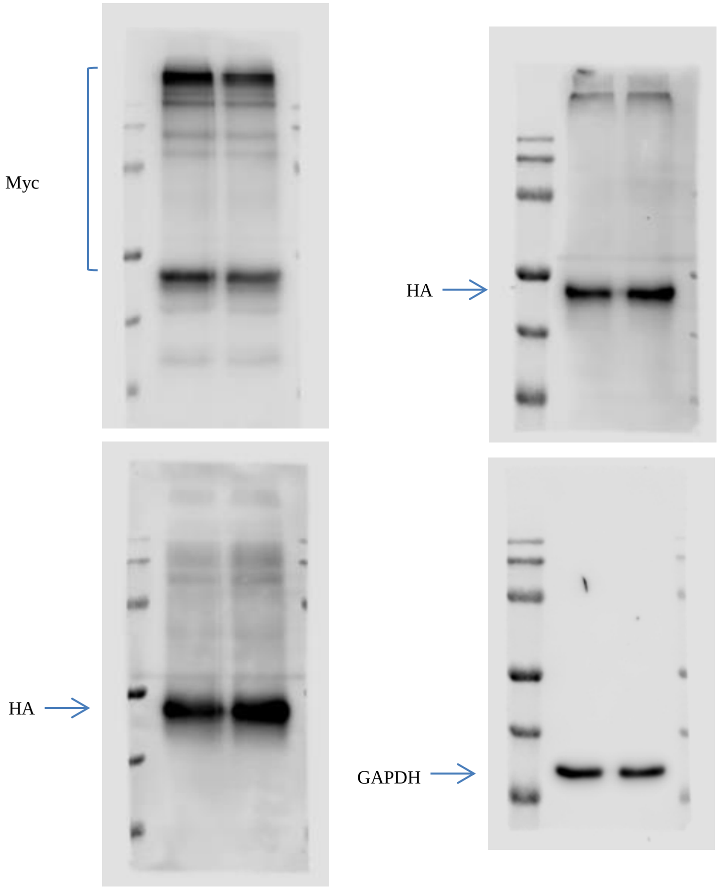

Full unedited gel for Supplementary Figure 11C.

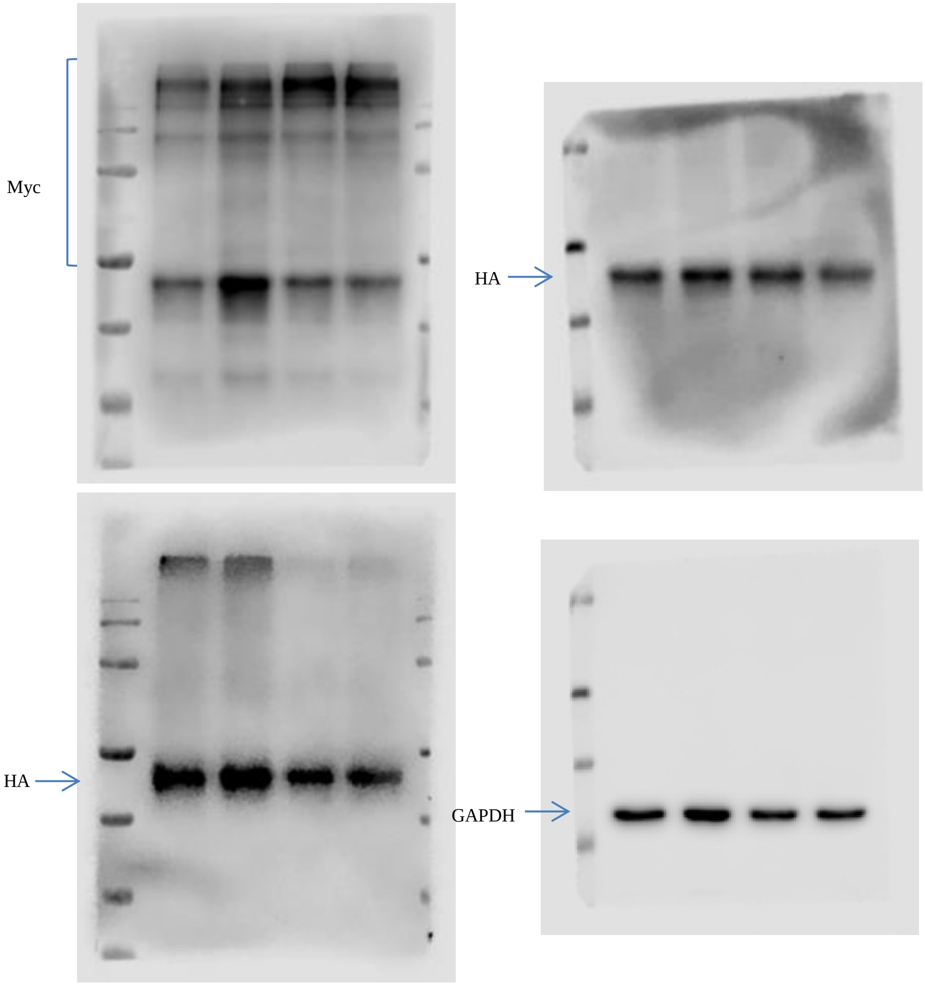

Full unedited gel for Supplementary Figure 11D.

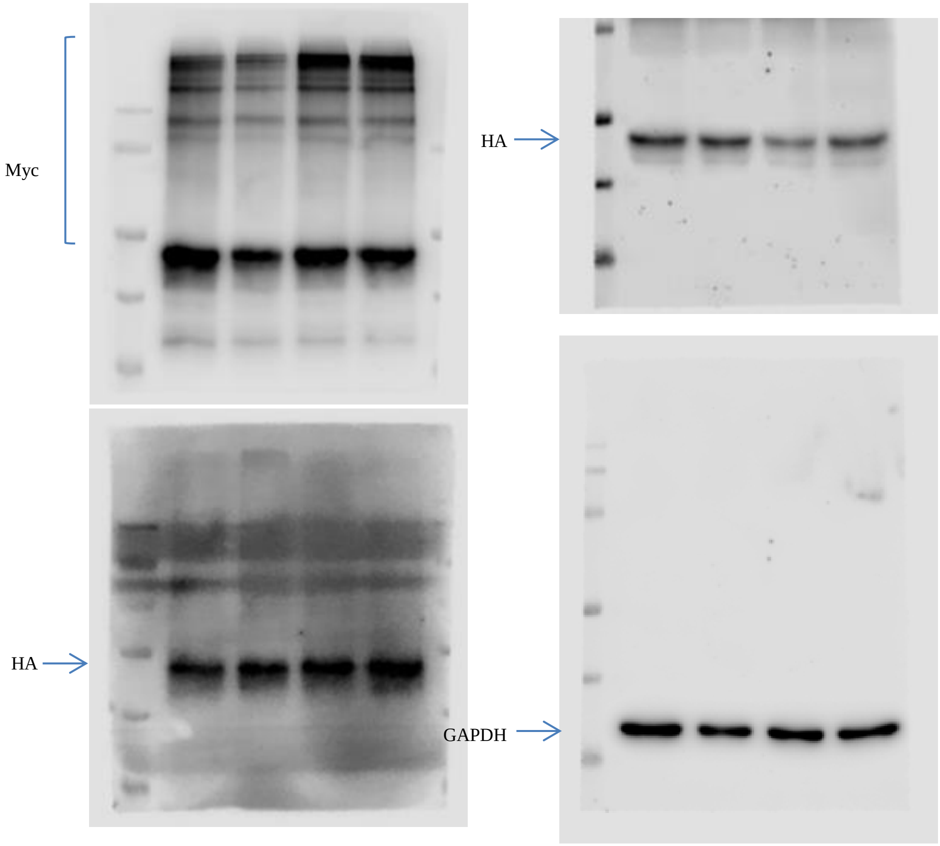

Supplement: Supplementary file 2 — unedited gel [file 41420_2023_1702_MOESM2_ESM.pdf]
